# Co-opting the fermentation pathway for tombusvirus replication: Compartmentalization of cellular metabolic pathways for rapid ATP generation

**DOI:** 10.1371/journal.ppat.1008092

**Published:** 2019-10-24

**Authors:** Wenwu Lin, Yuyan Liu, Melissa Molho, Shengjie Zhang, Longshen Wang, Lianhui Xie, Peter D. Nagy

**Affiliations:** 1 State Key Laboratory of Ecological Pest Control for Fujian and Taiwan Crops, Fujian Agriculture and Forestry University, Fuzhou, China; 2 Department of Plant Pathology, University of Kentucky, Lexington, Kentucky, United States of America; Agriculture and Agri-Food Canada, CANADA

## Abstract

The viral replication proteins of plus-stranded RNA viruses orchestrate the biogenesis of the large viral replication compartments, including the numerous viral replicase complexes, which represent the sites of viral RNA replication. The formation and operation of these virus-driven structures require subversion of numerous cellular proteins, membrane deformation, membrane proliferation, changes in lipid composition of the hijacked cellular membranes and intensive viral RNA synthesis. These virus-driven processes require plentiful ATP and molecular building blocks produced at the sites of replication or delivered there. To obtain the necessary resources from the infected cells, tomato bushy stunt virus (TBSV) rewires cellular metabolic pathways by co-opting aerobic glycolytic enzymes to produce ATP molecules within the replication compartment and enhance virus production. However, aerobic glycolysis requires the replenishing of the NAD^+^ pool. In this paper, we demonstrate the efficient recruitment of pyruvate decarboxylase (Pdc1) and alcohol dehydrogenase (Adh1) fermentation enzymes into the viral replication compartment. Depletion of Pdc1 in combination with deletion of the homologous *PDC5* in yeast or knockdown of Pdc1 and Adh1 in plants reduced the efficiency of tombusvirus replication. Complementation approach revealed that the enzymatically functional Pdc1 is required to support tombusvirus replication. Measurements with an ATP biosensor revealed that both Pdc1 and Adh1 enzymes are required for efficient generation of ATP within the viral replication compartment. In vitro reconstitution experiments with the viral replicase show the pro-viral function of Pdc1 during the assembly of the viral replicase and the activation of the viral p92 RdRp, both of which require the co-opted ATP-driven Hsp70 protein chaperone. We propose that compartmentalization of the co-opted fermentation pathway in the tombusviral replication compartment benefits the virus by allowing for the rapid production of ATP locally, including replenishing of the regulatory NAD^+^ pool by the fermentation pathway. The compartmentalized production of NAD^+^ and ATP facilitates their efficient use by the co-opted ATP-dependent host factors to support robust tombusvirus replication. We propose that compartmentalization of the fermentation pathway gives an evolutionary advantage for tombusviruses to replicate rapidly to speed ahead of antiviral responses of the hosts and to outcompete other pathogenic viruses. We also show the dependence of turnip crinkle virus, bamboo mosaic virus, tobacco mosaic virus and the insect-infecting Flock House virus on the fermentation pathway, suggesting that a broad range of viruses might induce this pathway to support rapid replication.

## Author summary

Replication of positive-strand RNA viruses, which infect plants and animals, depends on many cellular resources. These viruses subvert cellular membranes and co-opt host proteins to build replication compartments that produce the viral progeny. The viral replication process also requires cellular metabolites and energy in the form of ATP. Using plant host as well as the model host yeast, the authors discovered that tomato bushy stunt tombusvirus (TBSV) co-opts the fermentation pathway to facilitate the local generation of ATP within the replication compartments. TBSV replication induces high expression of pyruvate decarboxylase (Pdc1) and alcohol dehydrogenase (Adh1) fermentation enzymes. With the help of the viral replication protein, TBSV recruits Pdc1 and Adh1 into the viral replication compartment. The authors propose that compartmentalization of the fermentation pathway gives an evolutionary advantage for tombusviruses by facilitating rapid and efficient replication in the infected hosts. The authors also showed that the replication of five other plant viruses depends on the fermentation pathway in plants.

## Introduction

Similar to other positive-strand RNA viruses, the plant-infecting tombusviruses cause major structural rearrangements and metabolic changes in infected cells. The changes include the subversion of pro-viral host factors to support their replication and the induction of lipid synthesis, lipid transfer, membrane proliferation and alteration of vesicular trafficking. The major outcome of all these virus-driven processes is the biogenesis of the unique and extensive viral replication compartments and the formation of numerous viral replicase complexes (VRCs) on subverted subcellular membrane surfaces [1–7]. All these cellular changes serve several purposes, including supporting robust viral RNA replication, and protection of the viral RNA, including the dsRNA replication intermediate, from recognition by the cellular innate immune system or from elimination by the host RNAi machinery, which is also called post transcriptional gene silencing in plants [8–11]. Also, sequestration of viral and co-opted host proteins together with the viral (+)RNA into the replication compartment results in high local concentrations and efficient macromolecular assembly needed for the optimal formation of VRCs [6,12–19]. Our increasing knowledge of the roles of various lipids/membranes and co-opted host factors in RNA virus replication will be useful to control RNA viruses.

Tombusviruses, such as tomato bushy stunt virus (TBSV) and carnation Italian ringspot virus (CIRV), are small (+)RNA viruses, which can replicate in the surrogate model host yeast (*Saccharomyces cerevisiae*) [20–22]. Intensive genome-wide and proteome-wide research with the TBSV–yeast system has led to a catalog of host factors co-opted for viral RNA replication [4,22,23]. Induction of global phospholipid biosynthesis and redistribution of sterol and alteration of vesicular trafficking has been revealed to play major roles in the formation of VRCs and the activation of the viral-coded p92 RdRp [24–28]. All these subcellular changes are guided by the p33 replication protein, which is the master regulator of VRC assembly and viral (+)RNA recruitment into the VRCs [24,29]. Additional characteristic alterations caused by TBSV include the subversion of the actin network, the induction of subcellular membrane proliferation, peroxisome aggregation and the formation of membrane contact sites to support efficient virus replication [22,30,31].

Most of these viral-induced processes require ATP-based energy and the production of new metabolites in infected cells. Accordingly, we have previously discovered that several glycolytic enzymes, such as glyceraldehyde-3-phosphate dehydrogenase (GAPDH, Tdh2/3 in yeast), phosphoglycerate kinase (Pgk1) and pyruvate kinase (PK, Cdc19 in yeast) are recruited into the viral replication compartment [32–35] and Eno2 phosphopyruvate hydratase binds to p92^pol^ replication protein [27]. These findings suggest that tombusviruses co-opt the aerobic glycolytic pathway, which leads to the production of plentiful ATP within the viral replication compartment [32,33]. Our studies also revealed that the locally produced ATP is used up to fuel the co-opted Hsp70 proteins and DEAD-box helicases, and possibly the ESCRT-associated Vps4 AAA ATPase to promote viral replication within the viral replication compartment [32,33].

Sustaining aerobic glycolysis pathway, however, requires the replenishing of NAD^+^, which is a critical regulatory compound in glycolysis [36,37]. Because a previous proteomic-based screen indicated that p92^pol^ replication protein binds to Pdc1 pyruvate decarboxylase fermentation protein [27], in this work we studied the role of the fermentation pathway in tombusvirus replication.

Aerobic glycolysis occurs in many fast-growing microbes, and in cancer cells, some embryonic cells and immune cells, such as fibroblasts and lymphocytes [36–38]. Aerobic glycolysis is hijacked by the malaria parasite [36]. It is a process that regulates the balance between fast ATP production and biosynthesis of ribonucleotides, lipids and several amino acids. However, aerobic glycolysis requires the replenishing of NAD^+^ compound, which is produced by fermentation in eukaryotic cells. NAD^+^ is converted to NADH during glycolysis by GAPDH, which is required for many biosynthetic processes [36–38].

Our surprising discovery is that tombusviruses co-opt the host Pdc1 and the alcohol dehydrogenase (Adh1) fermentation enzymes by re-localizing them from the cytosol into the large viral replication compartment through direct interaction with the tombusvirus replication proteins. The subversion of the fermentation enzymes is critical to support VRC assembly, the activation of p92 RdRp protein and viral RNA synthesis. Most important, however, is the role of the co-opted fermentation enzymes in the maintenance of ATP synthesis within the viral replication compartment. Altogether, we discovered that tombusviruses compartmentalize entire cellular metabolic pathways to promote intensive viral replication within the viral replication compartment at the expense of the infected host cells. Based on these and previous findings, we propose that compartmentalization of the fermentation pathway gives an evolutionary advantage for tombusviruses to replicate quickly to speed ahead of antiviral responses of the hosts.

## Results

### Pdc1 fermentation enzyme is required for tombusvirus replication

**Fig 1:**
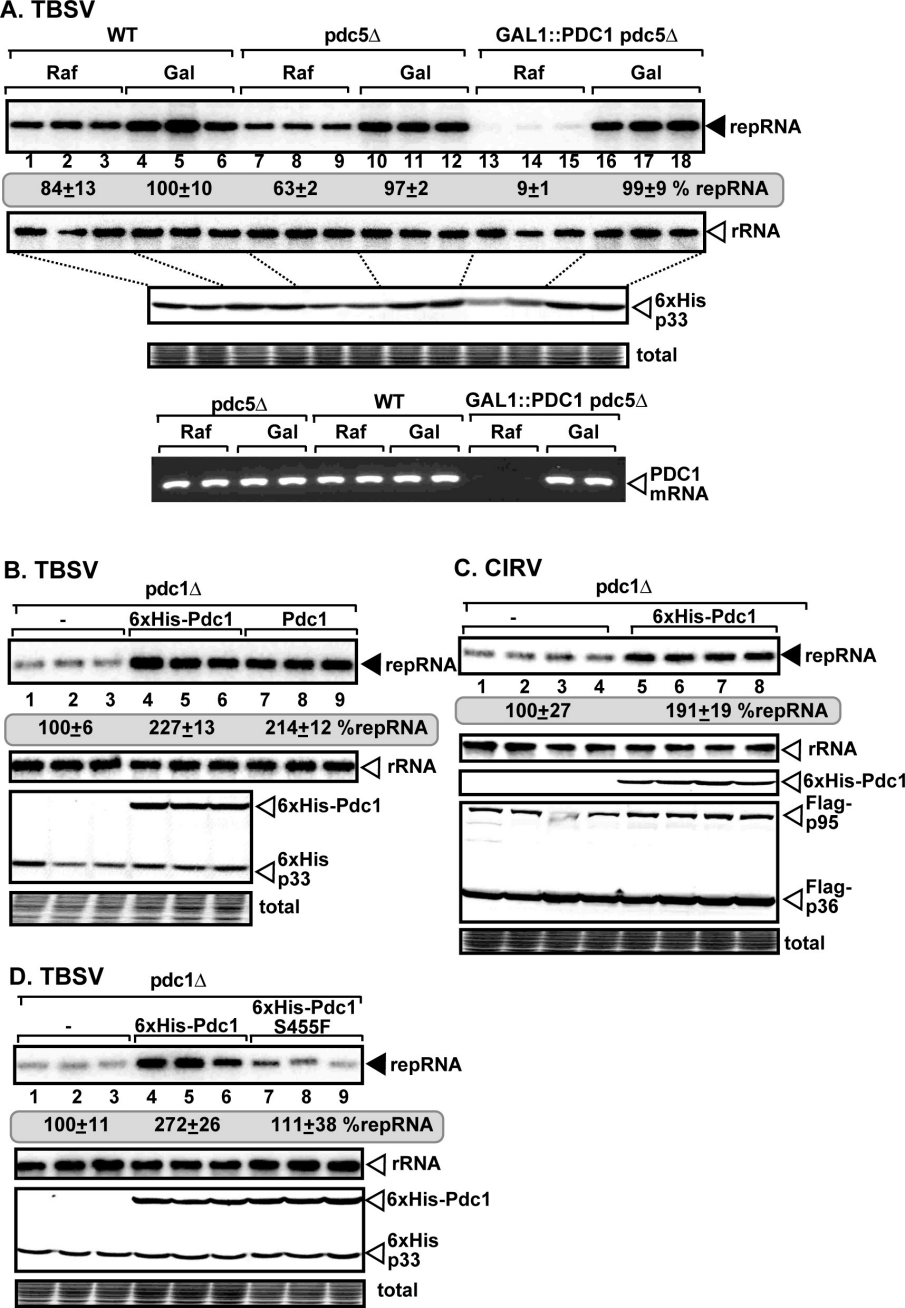
Pdc1 fermentation enzyme is an essential host factor for tombusvirus replication in yeast. (A) Depletion of pyruvate decarboxylase (Pdc1p) in combination with deletion of the homologous *PDC5* inhibits TBSV replicon (rep)RNA replication in yeast. Top panels: northern blot analyses of TBSV repRNA using a 3’ end specific probe demonstrates reduced accumulation of repRNA in GAL::PDC1 pdc5Δ yeast strain with depleted Pdc1p (raffinose-containing media) in comparison with the WT yeast strain or GAL::PDC1 pdc5Δ yeast strain with induced Pdc1p (galactose-containing media). Viral proteins His_6_-p33 and His_6_-p92 of TBSV were expressed from plasmids from the copper-inducible *CUP1* promoter, while DI-72(+) repRNA was expressed from the constitutive *TET1* promoter. Second panel: northern blot with an 18S ribosomal RNA specific probe was used as a loading control. Bottom images: western blot analysis of the level of His_6_-tagged p33 protein with anti-His antibody. Coomassie blue-stained SDS-PAGE was used for protein loading control. The down-regulation of Pdc1 mRNA was confirmed with RT-PCR. Each experiment was repeated three times. (B-D) Expression of Pdc1p from a plasmid increases tombusvirus replication in pdc1Δ yeast strain. His_6_-p33 and His_6_-p92 were expressed from the *GAL1* promoter, whereas (+)repRNA was expressed from the *GAL10* promoter. For panel C, Flag-p36 and Flag-p95 were expressed from the *CUP1* promoter, whereas the repRNA from the *GAL10* promoter. The untagged or His_6_-tagged Pdc1 were expressed from the *TET* promoter in all these experiments. See further details in panel A above.

Pdc1 was previously identified in a co-purification assay with the TBSV p92^pol^ replication protein [27]. To test the relevance of *PDC1* in tombusvirus replication, first we created a double mutant, which allowed for the depletion of Pdc1p in the absence of Pdc5p paralog in yeast (GAL::PDC1 pdc5Δ). Then, we used this double mutant yeast to launch TBSV replication via co-expressing the p33 and p92^pol^ replication proteins and the replicon repRNA. Northern blot analysis revealed a ~10-fold decrease in TBSV repRNA accumulation when Pdc1p expression was suppressed versus induced (Fig 1, lanes 13–15 versus 16–18). These data suggest that Pdc1p and Pdc5p are critical for TBSV replication. Expression of Pdc1p from a plasmid in pdc1Δ yeast increased TBSV repRNA accumulation by ~2-fold (Fig 1B). We observed similar ~2-fold enhanced replication of the closely-related CIRV, which replicates on the outer membranes of mitochondria (Fig 1C). Depletion of Pdc1p in double mutant yeast (GAL::PDC1 pdc5Δ) also inhibited the replication of the unrelated Flock House virus (FHV), which is an insect-infecting RNA virus (S1 Fig, lanes 17–20 versus 1–24). FHV replicates on the outer membranes of mitochondria [39]. These data suggest that Pdc1p has a pro-viral role in different subcellular microenvironments.

To test if the canonical enzymatic function of Pdc1p is needed for TBSV replication, we expressed Pdc1^S455F^ mutant, which has a reduced pyruvate decarboxylase activity [40], in pdc1Δ yeast replicating TBSV repRNA. Unlike the WT Pdc1p, Pdc1^S455F^ mutant was unable to enhance the replication of TBSV repRNA (Fig 1D, lanes 7–9 versus 4–6). Therefore, we suggest that the canonical role of Pdc1p in the fermentation pathway is required for efficient TBSV replication in yeast.

To obtain additional evidence for the pro-viral role of Pdc1p, we replaced the original promoter of *PDC2* gene with the regulatable *GAL1* promoter in the haploid yeast chromosome. Pdc2p is a transcription factor regulating the transcription of both *PDC1* and *PDC5* genes in yeast [41]. Depletion of Pdc2p in the above yeast (GAL1::HA-PDC2) resulted in ~3-fold reduction in TBSV repRNA accumulation (S2 Fig, lanes 13–16 versus 17–24). Therefore, these findings confirm the pro-viral role of Pdc1/5p in TBSV replication in yeast.

### Pdc1 protein interacts with the tombusvirus replication proteins

**Fig 2:**
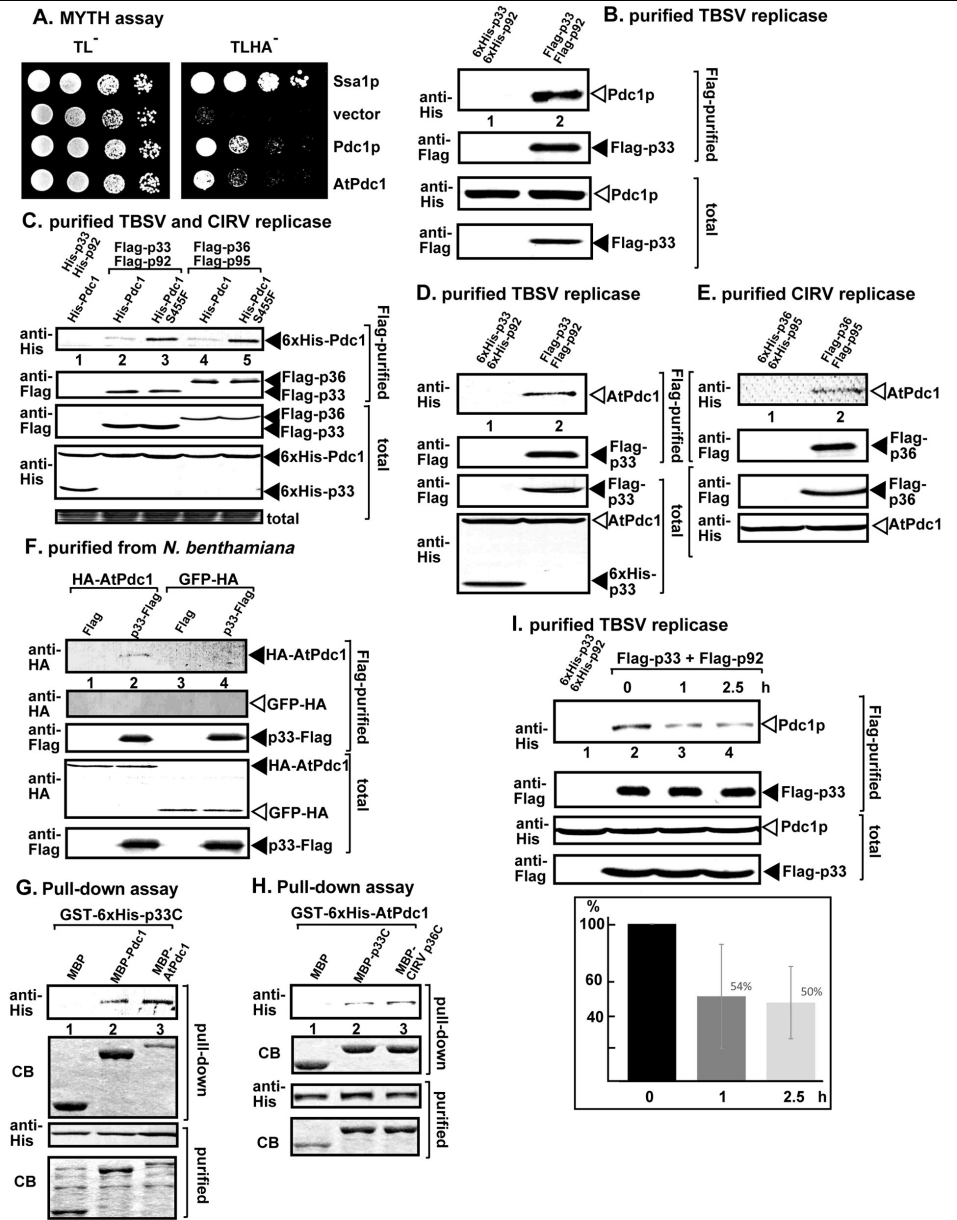
Interaction between tombusvirus replication proteins and Pdc1 fermentation enzyme. (A) The split ubiquitin-based MYTH assay was used to test binding between the TBSV p33 and the yeast Pdc1p and *Arabidopsis* Pdc1 proteins in yeast. The bait p33 was co-expressed with the shown prey proteins. The Ssa1p heat shock protein 70 (Hsp70) and the empty prey vector (NubG) were used as the positive and the negative controls, respectively. The right panel shows p33: Pdc1 interactions, the left panel demonstrates that comparable amounts of yeasts were used for these experiments. (B-C) Co-purification of the yeast His_6_-Pdc1p and His_6_-Pdc1^S455F^ mutant with TBSV Flag-p33 and Flag-p92^pol^ or the carnation Italian ringspot virus (CIRV) Flag-p36/Flag-p95 replication proteins from subcellular membranes. Top two panels: western blot analysis of the co-purified WT His_6_-Pdc1p and His_6_-Pdc1^S455F^ mutant with the Flag-affinity purified replication proteins. The His_6_-tagged proteins were detected with an anti-His antibody, while Flag-p33 and Flag-p36 were detected with an anti-Flag antibody. The negative control was from yeast expressing His_6_-p33 and His_6_-p92^pol^ purified using a Flag-affinity column (lane 1). Samples were cross-linked with formaldehyde in intact yeast cells. Bottom two panels: western blot of total His_6_-Pdc1p and Flag-p33 and Flag-p36 in the total yeast extracts. (D-E) Co-purification of the *Arabidopsis* His_6_-Pdc1 with the TBSV Flag-p33 and Flag-p92^pol^ or the CIRV Flag-p36/Flag-p95 replication proteins from subcellular membranes of yeast. Top two panels: western blot analysis of the co-purified His_6_-AtPdc1 with Flag-affinity purified replication proteins. The His_6_-tagged proteins were detected with anti-His antibody, while the Flag-p33 and Flag-p36 were detected with an anti-Flag antibody. The negative control was from yeast expressing the His_6_-tagged replication proteins purified using a Flag-affinity column (lane 1). Samples were cross-linked with formaldehyde. Bottom two panels: western blot of the total Flag-p33 or Flag-p36 and the His_6_-AtPdc1 and in the total yeast extracts (F) Co-purification of HA-AtPdc1 with the TBSV p33-Flag replication protein from *N*. *benthamiana*. Top two panels: western blot analysis of the co-purified HA-tagged AtPdc1 (lane 2) with the Flag-affinity purified Flag-p33. HA-Pdc1 was detected with an anti-HA antibody, while the p33-Flag was detected with an anti-Flag antibody as shown. Bottom two panels: western blot of the total plant extracts. (G) Pull-down assay including the GST-His_6_-p33 replication protein and the MBP-tagged yeast Pdc1p or the MBP-AtPdc1. Note that we used the soluble C-terminal region of the TBSV p33 replication protein, which lacked the N-terminal sequence, including the trans-membrane TM domain. Top panel: western blot analysis of the captured His_6_-p33 with the MBP-affinity purified MBP-Pdc1 was performed with an anti-His antibody. The negative control was MBP (lane 1). Middle panel: Coomassie-blue stained SDS-PAGE of the captured yeast MBP-Pdc1p or MBP-AtPdc1 and MBP. Bottom panels: western blot analysis of the His_6_-p33 in the total extracts. Coomassie-blue stained SDS-PAGE of the MBP-Pdc1p or MBP-AtPdc1 and MBP in the total extracts. Each experiment was repeated three times. (H) Pull-down assay including the GST-His_6_-AtPdc1 and the MBP-tagged p33 or the CIRV MBP-p36 replication proteins. Please see further details in panel G. (I) Decreasing level of co-purification of His_6_-Pdc1p with the Flag-tagged viral replicase after blocking new VRC assembly. The yeast samples were collected at the shown time points after the addition of cycloheximide (blocks cellular translation, thus new VRC formation) to the yeast culture. Note that samples were from yeasts replicating TBSV repRNA. Top panel: western blot analysis of the co-purified His_6_-Pdc1with the Flag-affinity purified Flag-p33 and Flag-p92^pol^ from membrane fraction of yeast. The His_6_-Pdc1p was detected with an anti-His antibody. The negative control was the His_6_-p33 and His_6_-p92^pol^ purified from yeast extracts using a Flag-affinity column. Middle panel: western blot of the purified Flag-p33 detected with an anti-Flag antibody. Bottom panels: western blots of His_6_-Pdc1p and Flag-p33 proteins in the total yeast extracts using an anti-His and an anti-Flag antibodies. The graph shows the % of co-purified His_6_-Pdc1p with the tombusviral replication proteins with standard deviation. Each experiment was repeated three times.

To test if Pdc1p could interact with the tombusvirus replication proteins, we used the membrane yeast two-hybrid assay (MYTH), which is based on the split-ubiquitin strategy [42]. We found that the yeast Pdc1p and the homologous *Arabidopsis* AtPdc1 proteins interacted with the TBSV p33 replication protein (Fig 2A).

We then purified the TBSV replicase from yeast membrane fraction through detergent-solubilization and Flag-affinity purification. Interestingly, the yeast Pdc1p was co-purified with the Flag-p33/Flag-p92 replication proteins (Fig 2B). We also found that the enzymatically inactive Pdc1^S455F^ mutant was co-purified with the TBSV replicase from yeast (Fig 2C, lane 3). The mitochondrial CIRV Flag-p36/Flag-p95 showed a comparable co-purification profile with the WT Pdc1p and Pdc1^S455F^ mutant to that observed with the TBSV replication proteins (Fig 2C). The homologous AtPdc1 was also co-purified with either TBSV Flag-p33/Flag-p92 or the CIRV Flag-p36/Flag-p95 replication proteins from the mebrane-fraction of yeast (Fig 2D and 2E). Similar co-purification experiments with the Flag-p33 replication protein from detergent-solubilized fraction of *Nicotiana benthamiana* also confirmed the interaction of the replication proteins with AtPdc1 protein (Fig 2F). These data suggest that the interaction between Pdc1/AtPdc1 and p33 replication protein occurs in both yeast and plant cells.

To confirm direct interactions between the TBSV p33 and Pdc1p, we applied a pull-down assay with MBP-tagged Pdc1p or MBP-AtPdc1 and GST-His_6_-tagged p33C (the C-terminal, soluble portion) proteins from *E*. *coli* (Fig 2G). Both MBP-Pdc1p and MBP-AtPdc1 captured the GST-His_6_-p33C protein on the maltose-column, indicating direct interaction between these host and viral proteins. This conclusion was confirmed using the TBSV MBP-p33C or the CIRV MBP-p36C proteins, which captured the GST-His_6_-AtPdc1 in the second pull-down assay (Fig 2H). In the pull-down assay, we used truncated TBSV p33 and CIRV p36 replication proteins missing their membrane-binding regions to aid their solubility in *E*. *coli* (Fig 2G and 2H). Altogether, these data suggest that the direct interactions between the replication proteins of TBSV and CIRV and Pdc1p/AtPdc1 host proteins occur within the viral protein C-terminal domain facing the cytosolic compartment.

To examine if Pdc1p was co-opted as a permanent or temporary component of the tombusvirus replicase, first, we stopped the formation of new tombusvirus replicase complexes by blocking ribosomal translation via adding cycloheximide to the yeast growth media [43]. Second, we performed Flag-affinity-purification of the tombusvirus replicase from the membrane fraction of yeast at various time-points. Interestingly, the amount of the co-purified Pdc1p was decreased by ~50% in the purified replicase preparations at the 2.5 h time point (Fig 2I, lanes 3–4 versus 2). The reduction of Pdc1p amount suggests that Pdc1p is likely released from the replicase. The release of Pdc1p likely occurs before the final assembly of the viral replicase, which ultimately forms a rather closed vesicle-like structure during replication [31,43,44]. Based on this observation, we suggest that the function of Pdc1p is temporary with the replication proteins, which likely takes place during the early steps of tombusvirus replication.

### The Adh1 family of fermentation enzymes is co-opted for tombusvirus replication

The fermentation pathway consists of two different sets of enzymes, pyruvate decarboxylase (Pdc1/5 in yeast) and alcohol dehydrogenase (Adh1-5 in yeast). The end result of the pathway is ethanol, however, the critical product is NAD^+^ from NADH [36,38]. Importantly, NAD^+^ is required to replenish the glycolytic pathway via providing the regulatory compound of Glyceraldehyde-3-phosphate dehydrogenase (GAPDH, coded by *TDH2/3* in yeast). Because the subversion of the catalytically active Pdc1 enzyme is needed to support TBSV replication (see above), we tested if the NAD^+^ producing Adh family members are also co-opted by tombusviruses.

**Fig 3:**
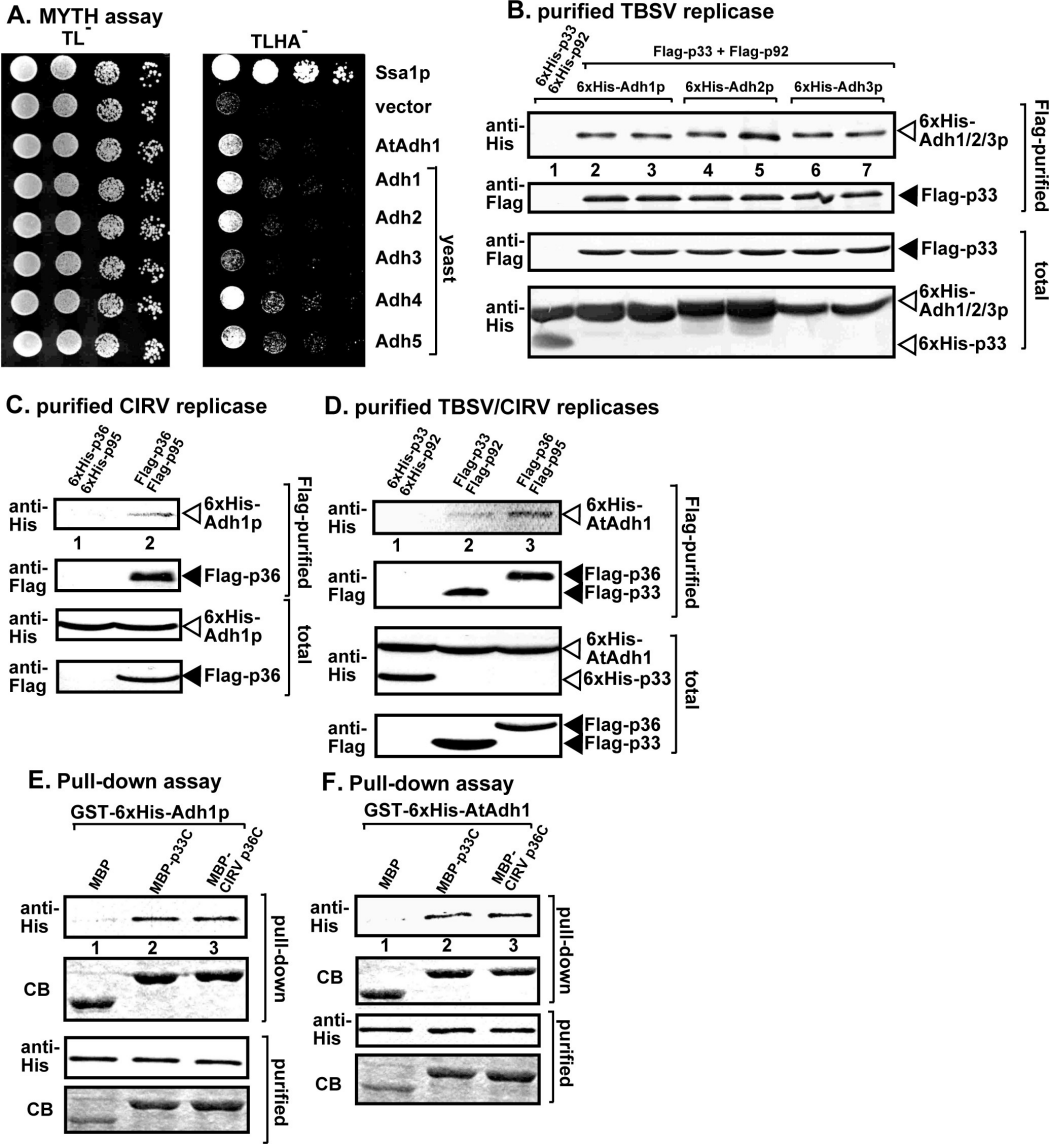
Interaction between the tombusvirus replication proteins and Adh1 fermentation enzyme. (A) The split ubiquitin-based MYTH assay was used to test binding between the TBSV p33 and the yeast alcohol dehydrogenase Adh1-5p and the *Arabidopsis* Adh1 proteins in yeast. The bait p33 was co-expressed with the shown prey proteins. The Ssa1p Hsp70 and the empty prey vector (NubG) were used as the positive and the negative controls, respectively. The right panel shows p33: Adh1-5 interactions, the left panel demonstrates that comparable amounts of yeasts were used for these experiments. (B) Co-purification of the yeast His_6_-Adh1, 2, 3p with the TBSV Flag-p33 and Flag-p92^pol^ replication proteins from subcellular membranes. Top two panels: western blot analysis of the co-purified His_6_-Adh1-3p with the Flag-affinity purified replication proteins. His_6_-tagged proteins were detected with an anti-His antibody, while Flag-p33 was detected with an anti-Flag antibody. The negative control was from yeast expressing His_6_-p33 and His_6_-p92^pol^ purified using a Flag-affinity column (lane 1). Samples were cross-linked with formaldehyde. Bottom two panels: western blot of the total His_6_-Adh1-3p and Flag-p33 in the total yeast extracts. (C) Co-purification of the yeast His_6_-Adh1p with the CIRV Flag-p36 and Flag-p95^pol^ replication proteins from subcellular membranes. See further details in panel B. (D) Co-purification of His_6_-AtAdh1 with either the TBSV or the CIRV replicase from yeast subcellular membranes. Top two panels: western blot analysis of the co-purified His_6_-Adh1p (lanes 2–3) with the Flag-affinity purified Flag-p33 or CIRV Flag-p36. His_6_-Adh1p was detected with an anti-His antibody, while the Flag-p33 or CIRV Flag-p36 replication proteins were detected with an anti-Flag antibody as shown. Bottom two panels: western blot of the total plant protein extracts. (E-F) Pull-down assay including GST-His_6_-Adh1p or GST-His_6_-AtAdh1 with the TBSV MBP-p33C or the CIRV MBP-p36C replication proteins and MBP. See further details in panel B. Each experiment was repeated three times.

To test if Adh1-5p could interact with the tombusvirus replication proteins, we used the MYTH assay [42], which revealed that the five members of the yeast Adh family as well as the homologous *Arabidopsis* AtAdh1 protein interacted with the TBSV p33 replication protein (Fig 3A). To confirm this unexpected finding, we purified the TBSV replicase from yeast membrane fraction through detergent-solubilization and Flag-affinity purification. We found that the yeast Adh1p, Adh2p and Adh3p were all co-purified with the Flag-p33/p92 replication proteins (Fig 3B). Adh1p was also co-purified with the mitochondrial CIRV Flag-p36/p95 replication proteins (Fig 3C), suggesting that different tombusviruses recruit Adh proteins into the membrane fraction of yeast. Similar co-purification experiments with the TBSV Flag-p33 or the CIRV Flag-p36 replication proteins from detergent-solubilized membranous fraction of yeast confirmed the interactions of the TBSV and CIRV replication proteins with AtAdh1 protein (Fig 3D). These data suggest that the interactions between Adh1/AtAdh1 and the tombusviral replication proteins occur in yeast cells.

To confirm direct interactions between TBSV p33 and Adh1p, we applied a pull-down assay using the TBSV MBP-p33C or the CIRV MBP-p36C proteins, which captured the GST-His_6_-Adh1p in the pull-down assay (Fig 3E). Similar pull-down experiment also confirmed the direct interaction between GST-His_6_-AtAdh1 and the viral replication proteins (Fig 3F). As above, we used the truncated TBSV p33 and CIRV p36 replication proteins missing their membrane-binding regions to increase their solubility in *E*. *coli* in the pull-down assay (Fig 3E–3F). Altogether, these data suggest that the direct interactions between the replication proteins of TBSV and CIRV and the Adh1 host protein occur within the viral protein domain facing the cytosol.

### Both Pdc1 and Adh1 have pro-viral functions in plants

**Fig 4:**
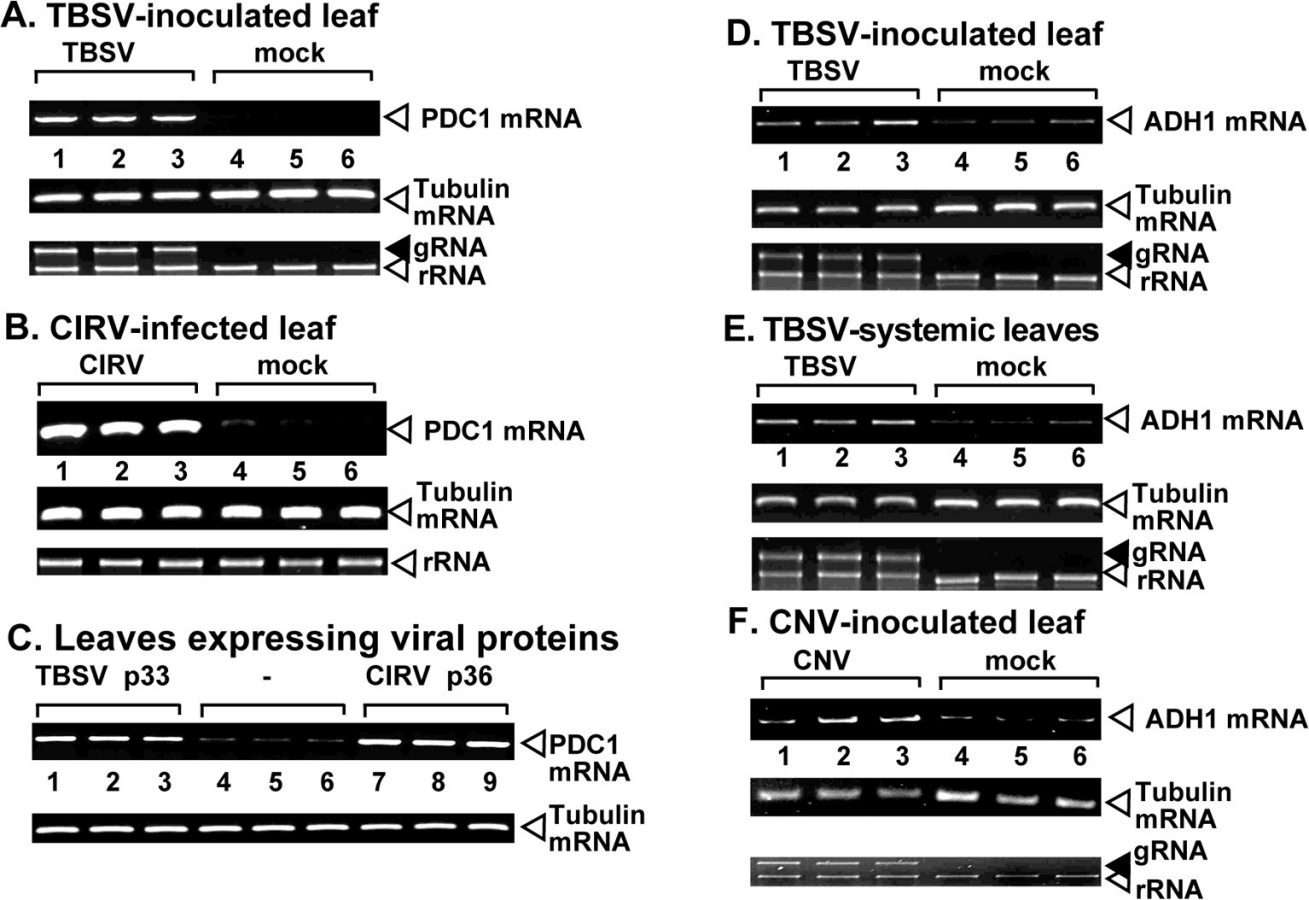
Tombusvirus infection induces the expression of Pdc1 and Adh1 mRNAs in *N*. *benthamiana*. (A) Top panels: semi-quantitative RT-PCR analysis of the NbPdc1 mRNA level at 1.5 dpi in *N*. *benthamiana* leaves infected with either TBSV or mock-infected. (B) Semi-quantitative RT-PCR analysis of the NbPdc1 mRNA level at 3 dpi in *N*. *benthamiana* leaves infected with either CIRV or mock-inoculated. The samples were taken 3 days after tombusvirus inoculation. Second panel: RT-PCR analysis of the tubulin mRNA level in the same plants. Each experiment was repeated three times. Bottom panels: Ethidium-bromide-stained agarose gels show the comparable amounts of RNA loading, as shown for the ribosomal RNA. (C) Top panel: semi-quantitative RT-PCR analysis of the NbPdc1 mRNA level at 3 days post agroinfiltration in *N*. *benthamiana* leaves agro-infiltrated to express TBSV p33, CIRV p36 or no-expression control. See further details in panels A-B. (D-F) Semi-quantitative RT-PCR analysis of the NbAdh1 mRNA level at 1.5 dpi in *N*. *benthamiana* leaves infected with either TBSV or mock-infected or CNV- or mock-infected at 3 dpi. See further details in panels A-B.

The homologous *PDC1* and *ADH1* genes are present in plants, but they are expressed at a very low level in plant cells under normal growth conditions [45,46]. Therefore, tombusviruses likely need to induce the expression of Pdc1 and Adh1 mRNAs in order to exploit Pdc1 and Adh1 for pro-viral functions during plant infections. Indeed, RT-PCR analysis of Pdc1 mRNA levels in TBSV-infected versus mock-treated *N*. *benthamiana* leaves revealed robust up-regulation of Pdc1 mRNA level in the TBSV inoculated leaves (Fig 4A) as well as the leaves expressing only the p33 replication protein (Fig 4C, lanes 1–3 versus 4–6). We observed a comparable up-regulation of Pdc1 mRNA level in CIRV infected *N*. *benthamiana* leaves or the leaves only expressing the CIRV p36 replication protein (Fig 4B and 4C). Based on these observations, we propose that TBSV and CIRV replication induces a high level of Pdc1 expression.

Similarly, RT-PCR analysis of Adh1 mRNA level in TBSV-infected versus mock-treated *N*. *benthamiana* leaves revealed an up-regulation of Adh1 mRNA level in the TBSV and CNV inoculated leaves (Fig 4D–4F) and the systemically-infected leaves (Fig 4E). Based on these observations, we propose that TBSV and CNV replication induces a high level of Adh1 expression in plant leaves.

**Fig 5:**
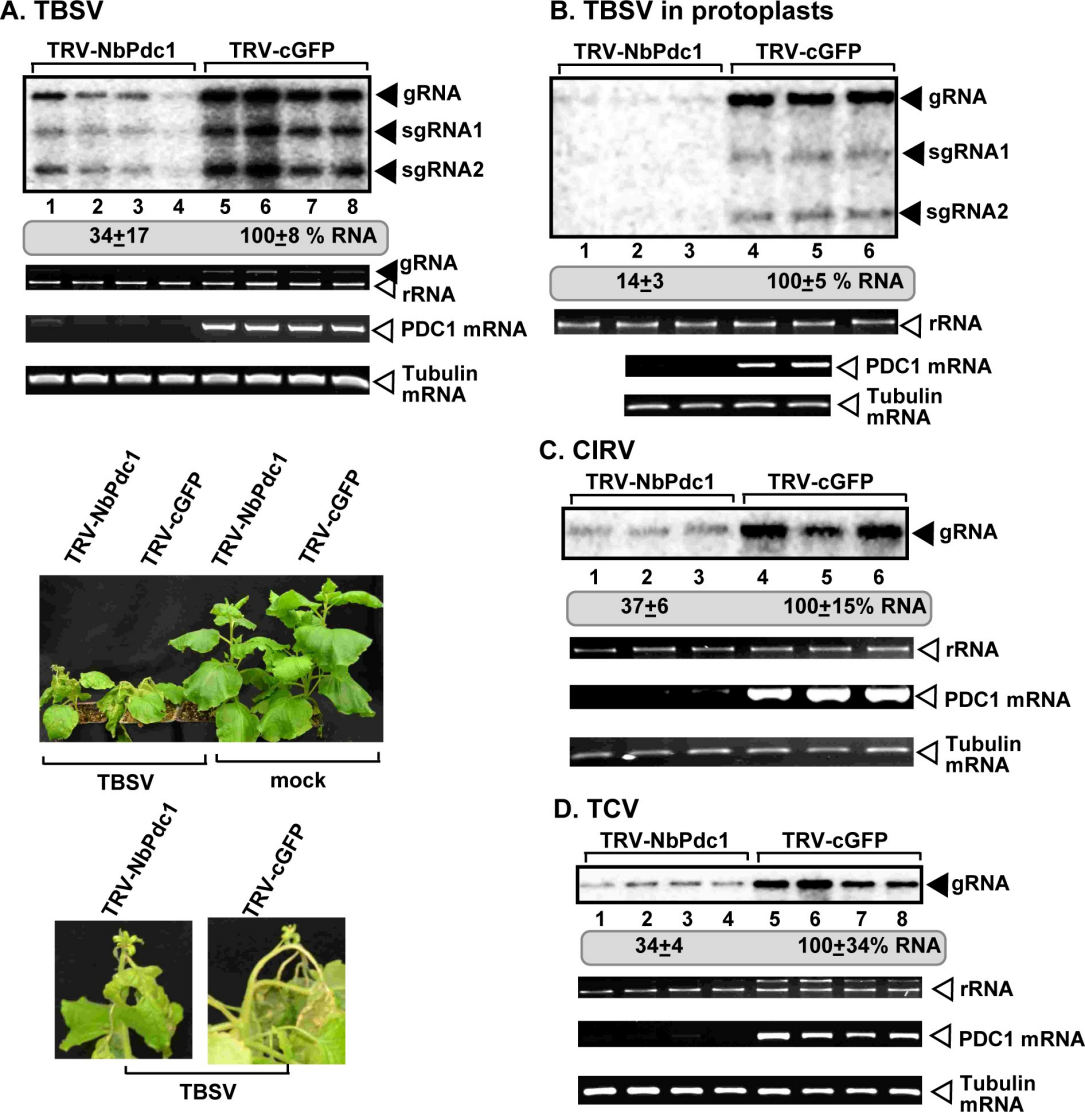
Knockdown of Pdc1 mRNA level inhibits tombusvirus replication in *N*. *benthamiana* plants. (A) Top panel: Accumulation of the TBSV genomic (g)RNA in *Pdc1*-silenced *N*. *benthamiana* plants 1.5 days post-inoculation (dpi) in the inoculated leaves was measured by northern blot analysis. Inoculation of TBSV gRNA was done 12 days after silencing of Pdc1 expression. Agroinfiltration of tobacco rattle virus (TRV) vector carrying NbPdc1 or 3’-terminal GFP (as a control) sequences was used to achieve virus-induced gene silencing (VIGS). Second panel: Ribosomal RNA is shown as a loading control in an ethidium-bromide stained agarose gel. Third panel: RT-PCR analysis of NbPdc1 mRNA level in the silenced and control plants. Fourth panel: RT-PCR analysis of tubulin mRNA level in the silenced and control plants. Each experiment was repeated three times. Delayed development of TBSV-induced symptoms is observed in the Pdc1-silenced *N*. *benthamiana* plants as compared with the control plants. Note the lack of phenotype in the Pdc1-silenced and mock-inoculated *N*. *benthamiana* plants. Note the severe wilting and beginning stage of necrosis in the control TBSV-infected plant versus the lack of those symptoms in the Pdc1-silenced *N*. *benthamiana* plants. The pictures were taken at 8 dpi. (B) Top panel: Accumulation of the TBSV gRNA in protoplasts isolated from Pdc1-silenced *N*. *benthamiana* was measured by northern blot analysis 16 hours after virus transfection. Protoplasts were isolated 12 days after silencing of Pdc1 expression. Agroinfiltration of TRV-NbPdc1 or TRV-cGFP (as a control) was used to induce VIGS. Second panel: Ribosomal RNA is shown as a loading control in an ethidium-bromide stained agarose gel. Third panel: RT-PCR analysis of NbPdc1 mRNA level in the silenced and the control protoplasts. Fourth panel: RT-PCR analysis of tubulin mRNA level in the silenced and the control protoplasts. Each experiment was repeated three times. (C) Accumulation of the CIRV gRNA in the Pdc1-silenced *N*. *benthamiana* plants 3 dpi in the inoculated leaves and at 5 dpi in the systemically-infected leaves was measured by northern blot analysis. See further details in panel A. (D) Accumulation of the TCV gRNA in the Pdc1-silenced *N*. *benthamiana* plants 6 dpi in the inoculated leaves was measured by northern blot analysis. See further details in panel A.

To study if tombusviruses depend on the Pdc1 function in plants, we knocked-down Pdc1 expression via virus-induced gene-silencing (VIGS) in *N*. *benthamiana* plants. Knockdown of Pdc1 in *N*. *benthamiana* resulted in a ~3-fold reduction of TBSV RNAs in the inoculated leaves (Fig 5A). Knockdown of Pdc1 level did not cause an obvious phenotype in *N*. *benthamiana* (Fig 5A). To test the effect of Pdc1 depletion on virus accumulation in the absence of cell-to-cell spread, we also tested TBSV replication in Pdc1 knockdown protoplasts. Interestingly, TBSV RNA accumulation was reduced by ~7-fold in Pdc1 knockdown protoplasts in comparison with control protoplasts (Fig 5B), suggesting that Pdc1 affects the viral replication process.

Similar experiments with CIRV in Pdc1 knockdown *N*. *benthamiana* plants also revealed a ~3-fold reduced level of tombusvirus accumulation (Fig 5C). These data confirmed the pro-viral role of Pdc1 in supporting tombusvirus replication in plants. To test if the pro-viral function of Pdc1 is also exploited by a more distantly-related carmovirus, we measured the accumulation of turnip crinkle virus (TCV) in Pdc1 knockdown *N*. *benthamiana* plants. The accumulation of TCV RNAs decreased by ~3-fold in Pdc1 knockdown plants (Fig 5D). It seems that tombusviruses and a carmovirus can exploit Pdc1 functions to support viral replication.

**Fig 6:**
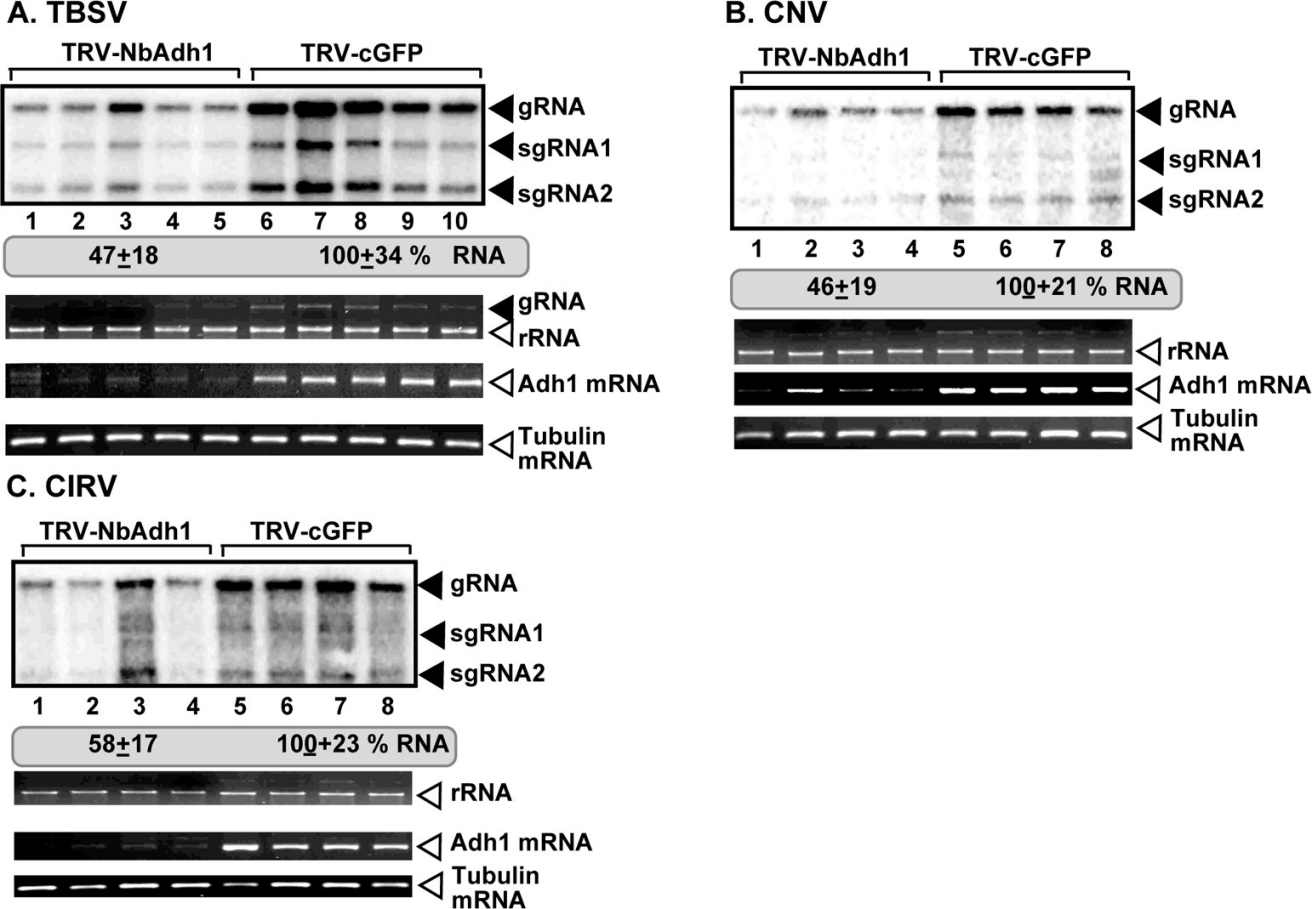
Knockdown of Adh1 mRNA level inhibits tombusvirus replication in *N*. *benthamiana* plants. (A-C) Accumulation of the TBSV, CNV and CIRV gRNA in the Adh1-silenced *N*. *benthamiana* plants. The experimental data are presented as in Fig 5.

Knockdown of Adh1 in *N*. *benthamiana* resulted in a ~2-fold reduction of TBSV RNAs in the inoculated leaves (Fig 6A). Knockdown of Adh1 in *N*. *benthamiana* also reduced the accumulation of the peroxisomal-replicating CNV and the mitochondrial-replicating CIRV by ~2-fold (Fig 6B and 6C). These data confirmed the pro-viral role of Adh1 in supporting tombusvirus replication in plants.

### Both Pdc1 and Adh1 proteins are recruited into the tombusvirus replication compartment in plants

**Fig 7:**
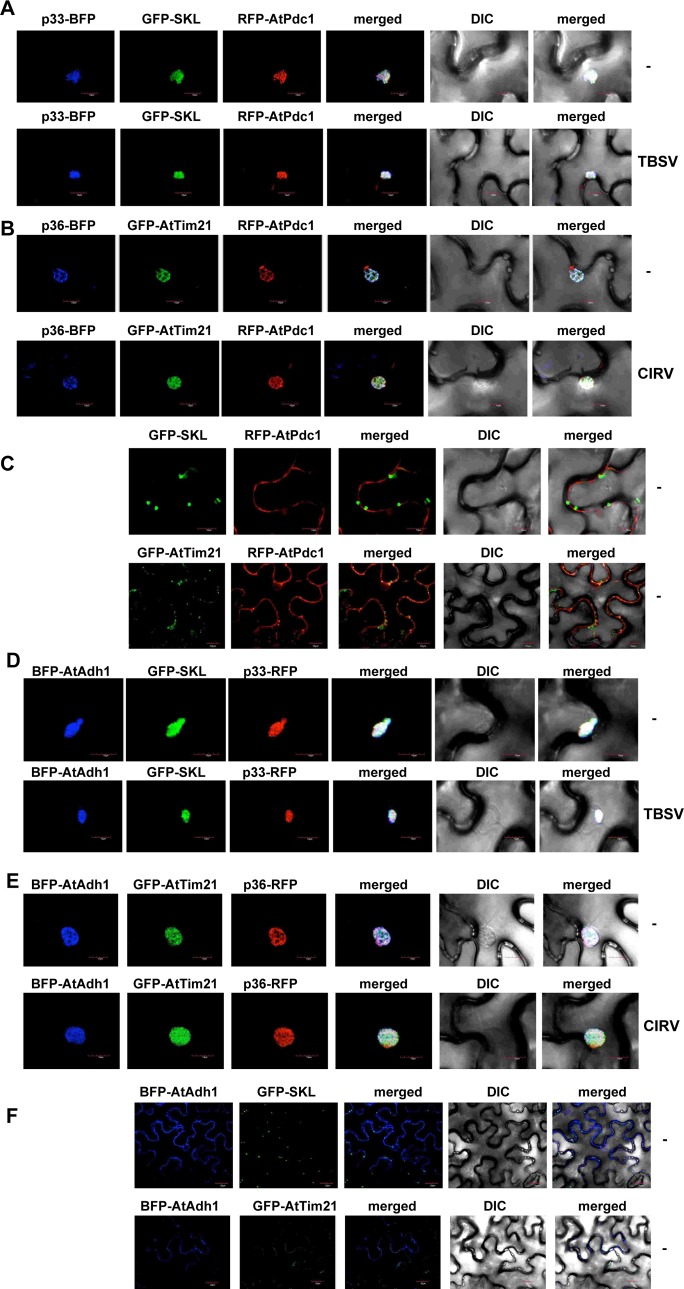
Recruitment of Pdc1 and Adh1 fermentation enzymes by the tombusvirus replication protein into the viral replication compartment in *N*. *benthamiana*. (A) Confocal microscopy images show efficient co-localization of the TBSV p33-BFP replication protein and the RFP-AtPdc1 within the viral replication compartment, marked by GFP-SKL peroxisomal luminal marker *in N*. *benthamiana* leaves. Expression of these proteins from the 35S promoter was done after co-agroinfiltration into *N*. *benthamiana* leaves. The plant leaves were either TBSV-infected or mock-inoculated as shown. The images were taken 1.5 days after TBSV inoculation of plant leaves. Scale bars represent 10 μm. (B) Recruitment of Pdc1 by the CIRV p36 replication protein into the mitochondria-derived viral replication compartment in *N*. *benthamiana*. Confocal microscopy images show efficient co-localization of CIRV p36-BFP replication protein and the RFP-AtPdc1 within the viral replication compartment, marked by GFP-AtTim21 mitochondrial marker *in N*. *benthamiana* leaves. The images were taken 1.5 days after agro-infiltration of plant leaves. See further details in panel A. (C) Confocal microscopy imaging shows the cytosolic localization of RFP-AtPdc1 in the absence of viral components. See further details in panel A. (D) Confocal microscopy images show efficient co-localization of TBSV p33-RFP replication protein and the BFP-AtAdh1 within the viral replication compartment, marked by GFP-SKL peroxisomal luminal marker *in N*. *benthamiana* leaves. See further details in panel A. (E) Recruitment of Adh1 by the CIRV p36 replication protein into the mitochondria-derived viral replication compartment in *N*. *benthamiana*. Confocal microscopy images show efficient co-localization of CIRV p36-RFP replication protein and the BFP-AtAdh1 within the viral replication compartment, marked by GFP-AtTim21 mitochondrial marker *in N*. *benthamiana* leaves. See further details in panel A. (F) Confocal microscopy imaging shows the cytosolic localization of BFP-AtAdh1 in the absence of viral components. See further details in panel A.

To determine if Pdc1 is recruited by TBSV into the extensive viral replication compartment, we co-expressed the BFP-tagged TBSV p33 replication protein and the RFP-tagged AtPdc1 with the GFP-SKL peroxisome matrix protein in *N*. *benthamiana* leaves, followed by confocal imaging. These experiments revealed a high level of co-localization of the TBSV p33 replication protein and the RFP-AtPdc1 within the replication compartments consisting of aggregated peroxisomes, even in the absence of TBSV replication (Fig 7A). We observed a similar re-distribution of the RFP-AtPdc1 in the presence of CIRV p36-BFP within the replication compartments consisting of aggregated mitochondria (Fig 7B). Therefore, we suggest that the TBSV p33 and the CIRV p36 replication proteins alone are enough to recruit Pdc1 to the replication compartment to a similar extent as the actively replicating TBSV or CIRV (Fig 7A and 7B). In the absence of viral components, AtPdc1 is localized in the cytosol (Fig 7C). Based on these experiments, we propose that Pdc1 is efficiently recruited by the tombusvirus replication proteins to the extensive tombusvirus replication compartments in plants.

Similar co-localization experiments revealed a high level of co-localization of the TBSV p33-RFP replication protein and the BFP-AtAdh1 within the replication compartments consisting of aggregated peroxisomes in the absence or presence of TBSV replication (Fig 7D). We also observed re-distribution of the BFP-AtAdh1 in the presence of CIRV p36-RFP within the replication compartments consisting of aggregated mitochondria (Fig 7E). Based on these observations, we suggest that the TBSV p33 and the CIRV p36 replication proteins alone are capable of recruiting AtAdh1 to the replication compartment (Fig 7). In the absence of viral components, AtAdh1 is localized in the cytosol (Fig 7F). These experiments support a model that Adh1 is efficiently recruited by the tombusvirus replication proteins to the extensive tombusvirus replication compartments in plants.

**Fig 8:**
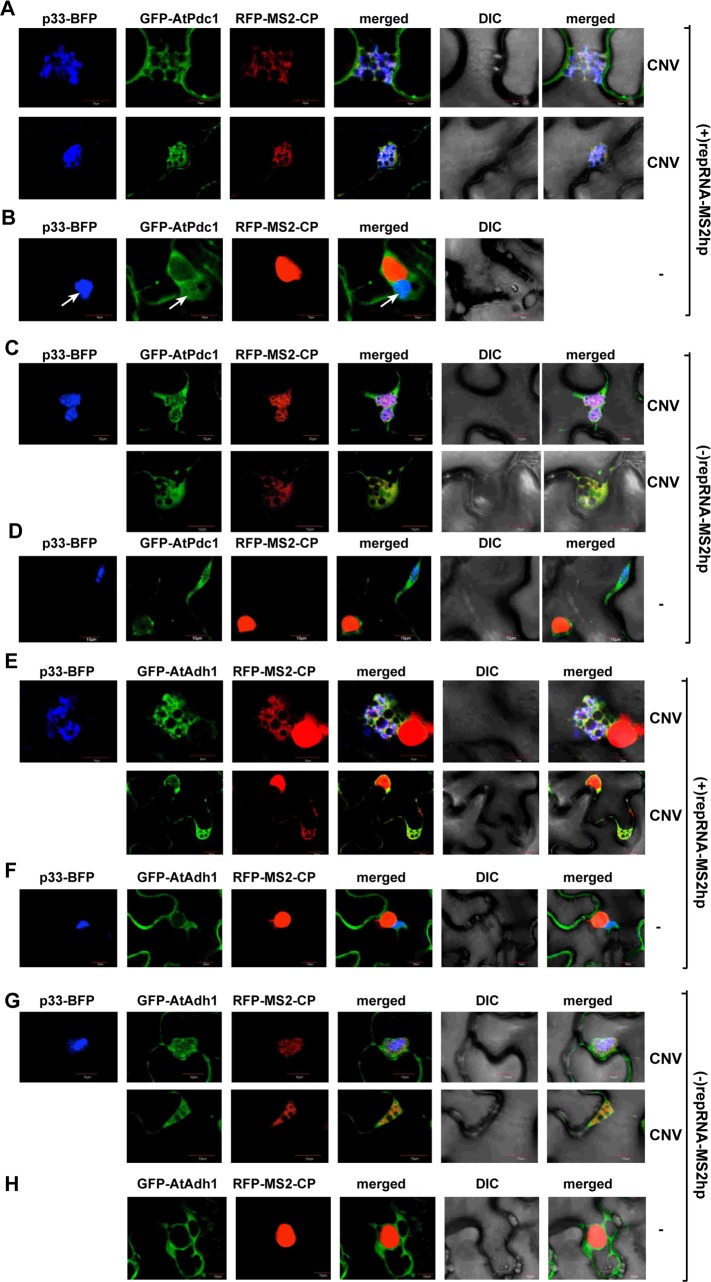
Confocal microscopy shows co-localization of the co-opted fermention enzymes with the viral repRNAs in whole plants infected with CNV. (A-B) Most of GFP-AtPdc1 is re-targeted into the replication compartment where the viral RNA synthesis takes place. The viral (+)repRNA carried six copies of the MS2 bacteriophage RNA hairpin (MS2hp), which is recognized by the MS2 coat protein (RFP-MS2-CP). The replication compartment was marked by the BFP-tagged p33 replication protein in *N*. *benthamiana*. Panel B shows images from plants mock-inoculated (no viral RNA replication). Note that RFP-MS2-CP contains a week nuclear localization signal, therefore this protein ends up in the nucleus in the absence of replicating (+)repRNA-MS2hp in the cytosol. Expression of the above proteins from the 35S promoter was done after co-agroinfiltration into *N*. *benthamiana* leaves. The images were taken 3.5 days after agro-infiltration of plant leaves. Scale bars represent 10 μm. Each experiment was repeated three times. (C-D) Similar experimental set-up as in panel A-B, except the six MS2hps form the suitable structures on the viral (-)repRNA-MS2hp, which is recognized by RFP-MS2-CP. See further details in Panel A. (E-H) Most of GFP-AtAdh1 is re-targeted into the replication compartment where the viral RNA synthesis takes place. See further details in Panel A and C.

To demonstrate whether AtPdc1 is recruited into the TBSV replication compartment, which actively replicates the viral RNAs, we utilized a modified repRNA carrying an ssRNA sensor [47]. This sensor consists of six repeats of a hairpin RNA from MS2 bacteriophage, which is specifically recognized by the MS2 coat protein (MS2-CP) [48]. Co-expression of the TBSV p33-BFP with the GFP-tagged AtPdc1 and the RFP-tagged MS2-CP revealed the re-localization of AtPdc1 to the active TBSV replication compartment containing the new (+)repRNA product (Fig 8A and S3A Fig) or the (-)repRNA, which is part of the replication intermediate (Fig 8C). In the control experiments, in the presence of only the TBSV repRNA and p33-BFP (no replication due to the absence of p92^pol^ replication protein), AtPdc1 was still localized in the viral replication compartment with p33, whereas RFP-MS2-CP was located in the nucleus (Fig 8B and 8D and S3B Fig). Therefore, we conclude that Pdc1 is present at the sites of tombusvirus replication and Pdc1 likely plays a role in the formation of the tombusvirus replication compartments.

Similar experiments with AtAdh1 revealed the re-localization of AtAdh1 to the active TBSV replication compartment containing the new (+)repRNA product (Fig 8E) or the (-)repRNA, which is part of the replication intermediate (Fig 8G and S3C Fig). In the control experiments, when only the TBSV repRNA and p33-BFP were expressed without the p92^pol^ replication protein, then AtAdh1 was still re-localized into the viral replication compartment with p33, but RFP-MS2-CP was located in the nucleus (Fig 8F and 8H and S3D Fig). Therefore, we conclude that AtAdh1, similar to AtPdc1, is present at the sites of active tombusvirus replication.

**Fig 9:**
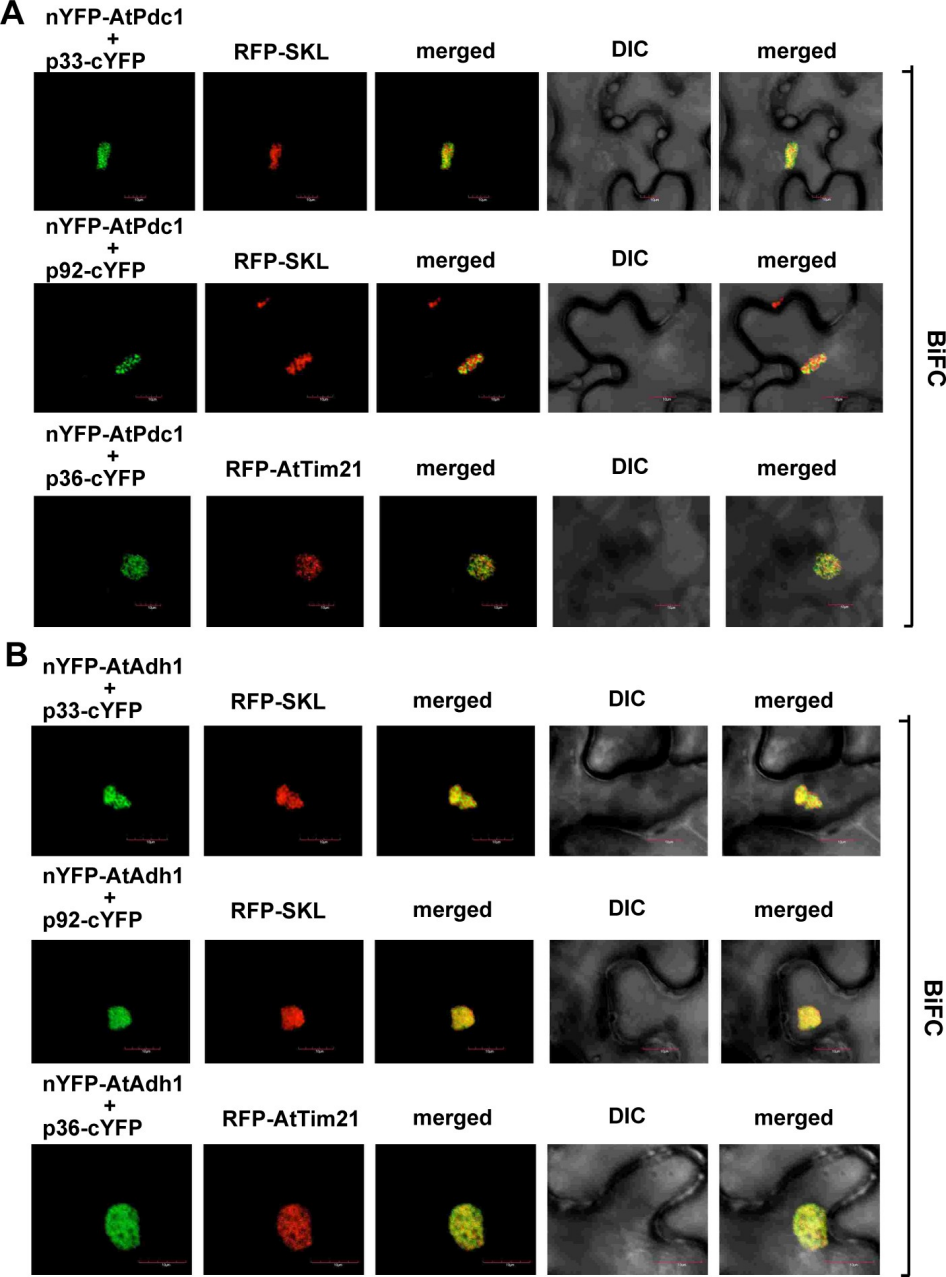
Interactions between TBSV p33/p92 or CIRV p36 replication proteins and the AtPdc1 or AtAdh1 proteins were detected by BiFC. The TBSV p33-cYFP or p92-cYFP or CIRV p36-cYFP replication proteins and the nYFP-AtPdc1 (panel A) or nYFP-AtAdh1 (panel B) proteins and the marker proteins were expressed via agroinfiltration. The merged images show the efficient co-localization of the peroxisomal RFP-SKL or the mitochondrial RFP-AtTim21 with the bimolecular fluorescence complementation (BiFC) signals, indicating that the interactions between the tombusvirus replication proteins and the co-opted AtPdc1 or AtAdh1 proteins occur in the large viral replication compartments, which consist of either aggregated peroxisomes or aggregated mitochondria. Scale bars represent 10 μm.

To provide additional evidence that the AtPdc1 is recruited into the viral replication compartments through interacting with the TBSV p33 or CIRV p36 replication proteins, we have conducted bimolecular fluorescence complementation (BiFC) experiments in *N*. *benthamiana* leaves. The BiFC experiments revealed robust interactions between AtPdc1 and either the TBSV p33/p92^pol^ or the CIRV p36 replication proteins within the replication compartment (Fig 9A, see also S4 Fig for the negative control BiFC experiments).

Similar BiFC experiments in *N*. *benthamiana* leaves revealed interactions between AtAdh1 and the TBSV p33/p92^pol^ or the CIRV p36 replication proteins within the replication compartment (Fig 9B). These data confirmed the replication protein-driven re-localization of AtPdc1 and AtAdh1 into the viral replication compartment.

### Pdc1 is required for efficient tombusvirus replication in vitro

**Fig 10:**
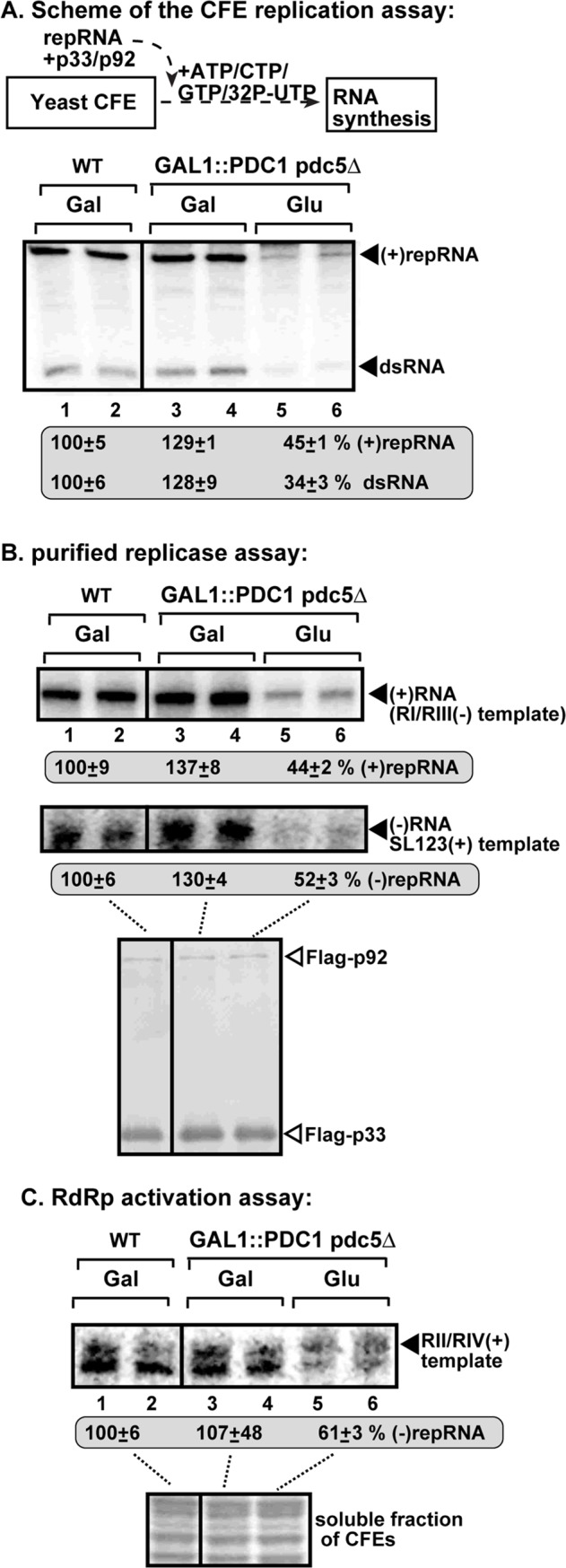
Dependence of TBSV repRNA accumulation on Pdc1/5 in an *in vitro* replicase reconstitution assay based on CFE obtained from yeast with depleted Pdc1/5. (A) Top: A scheme of the *in vitro* replicase reconstitution assay based on yeast cell-free extracts (CFEs). The purified recombinant TBSV p33 and p92^pol^ replication proteins from *E*. *coli* were added in combination with the (+)repRNA template to program the *in vitro* tombusvirus replication assay. The CFEs were prepared from yeast strains cultured in the shown media prior to CFE preparation. Bottom: Non-denaturing PAGE shows the accumulation of ^32^P-labeled (+)repRNAs and the dsRNA replication intermediate products made by the reconstituted replicases in the shown CFE preparations. All the samples shown were loaded on the same PAGE gel. Each experiment was repeated. (B) RdRp assay with Flag-affinity purified tombusvirus replicase preparations. The shown yeast strains expressing Flag-p33 and Flag-p92^pol^ from the *CUP1* promoter and (+)repRNA from the *TET* promoter were cultured in the shown media prior to preparation of the purified replicase preps. The replicase preparations containing the same amount of p33 replication protein were programmed with the shown (+) or (-)RNA templates. The denaturing PAGE gels show the produced complementary RNA products by the given replicase preparations. All the samples shown were loaded on the same PAGE gel. Each experiment was repeated. (C) The *in vitro* RdRp activation assay is based on (+)repRNA and p92-Δ167N RdRp protein in the presence of the soluble fraction of yeast CFE. The CFEs were prepared from yeast strains cultured in the shown media prior to CFE preparation. Denaturing PAGE analysis of the ^32^P-labeled RNA products obtained in an *in vitro* assay with recombinant p92-Δ167N RdRp. Each experiment was repeated three times.

To obtain direct evidence of the role of Pdc1 in TBSV replication, we used an in vitro replicase reconstitution assay based on a cell-free extract (CFE) from GAL::PDC1 pdc5Δ yeast strain with depleted Pdc1p level. Programming the CFE with the (+)repRNA and purified replication proteins led to ~3-to-4-fold reduced replication, including the production of both dsRNA replication intermediate and the (+)repRNA progeny when compared with CFE prepared from the same yeast strain with induced Pdc1p expression (Fig 10A). Based on these data, we suggest that Pdc1p is required for robust replication and likely during the replicase assembly step since all TBSV repRNA products were reduced when Pdc1p was depleted.

We also performed another approach to test the efficiency of replicase assembly in yeast, which is based on the purification of the tombusvirus replicase from yeast, followed by in vitro RdRp assay with added template RNA. The purified replicase prepared from GAL::PDC1 pdc5Δ yeast strain with depleted Pdc1p level had a reduced activity in comparison with the replicase obtained from the same yeast strain with induced Pdc1p expression on both (-) and (+)RNA templates (Fig 10B). Because the replicase has to pre-assemble in yeast in this approach, the reduced activity of replicase with depleted Pdc1p is likely due to defect in the replicase assembly step.

Interestingly, the replicase assembly involves the activation of the p92 RdRp, which depends on several viral- and host factors, most notably the co-opted Hsp70 protein chaperone [27]. We have tested the p92 RdRp activation in a simplified in vitro assay, based on a purified N-terminally-truncated p92 RdRp and the soluble fraction of yeast CFEs, which should provide the needed host components [27,28]. We observed a ~40% reduction in p92 RdRp activation when the CFE was derived from the double-mutant GAL::PDC1 pdc5Δ yeast strain with depleted Pdc1p level versus the CFE obtained from the same yeast strain with induced Pdc1p expression (Fig 10C). This reduction might indicate a low-level activity for the ATP-dependent Hsp70 in CFE, which could be due to reduced ATP production by glycolysis in yeast with a depleted Pdc1p level (see Discussion). Overall, all in vitro assays suggest the direct involvement of Pdc1 in tombusvirus replication, which is likely due to reduced ATP production by glycolysis.

### Robust generation of ATP by glycolysis in the tombusvirus replication compartment is dependent on the co-opted Pdc1 and Adh1 in yeast and plants

Because several pro-viral co-opted host proteins, such as Hsp70, the ESCRT-associated Vps4 AAA ATPase and DEAD-box helicases, require plentiful ATP within the replication compartment to fuel robust viral replication [43,49–53], it is possible that the co-opted Pdc1 and Adh1 are needed within the replication compartment to rapidly supply the NAD^+^ substrate. NAD^+^ is critical to replenish the glycolytic pathway, which is dependent on reducing NAD^+^ to NADH via the co-opted GAPDH (Tdh2/2p in yeast, GAPC in plants) [36,38]. The findings that both fermentation enzymes are recruited to the sites of virus replication and the catalytic activity of Pdc1 is required for its pro-viral function (Fig 1D), also support this hypothesis.

**Fig 11:**
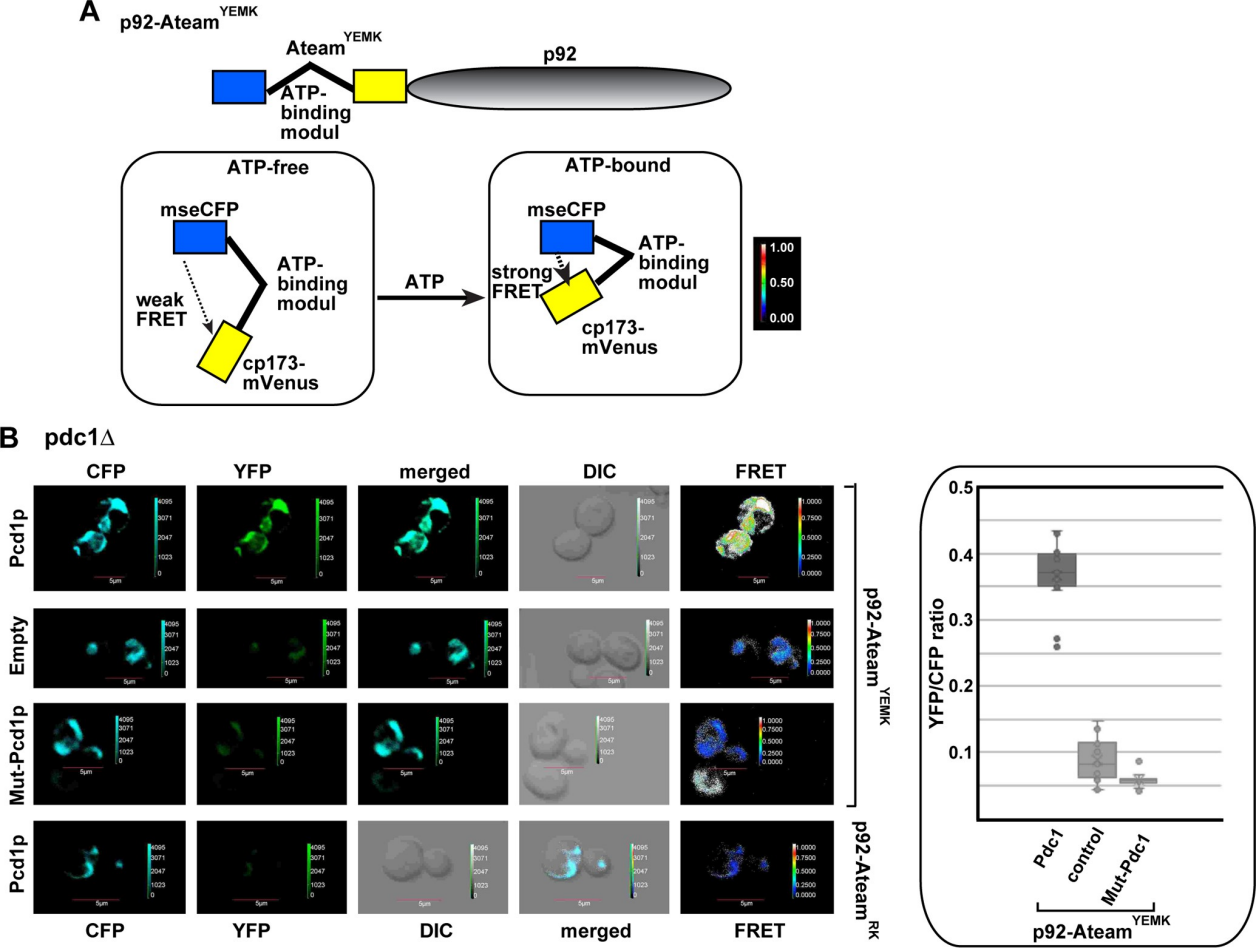
The co-opted cytosolic Pdc1 fermentation enzyme affects ATP accumulation within the tombusvirus replication compartment in yeast. (A) A scheme of the FRET-based detection of ATP within the tombusvirus replication compartment. The enhanced ATP biosensor, ATeam^YEMK^ was fused to TBSV p92^pol^ replication protein. See further details in the main text. (B) Comparison of the ATP level produced within the tombusvirus replication compartment in pdc1Δ yeast strain expressing WT Pdc1p, Pdc1^S455F^ mutant or without Pdc1 expression using ATeam^YEMK^ -p92^pol^. The more intense FRET signals are white and red (between 0.5 to 1.0 ratio), whereas the low FRET signals (0.1 and below) are light blue and dark blue. We show the quantitative FRET values (obtained with ImageJ) for a number of samples in the graph. Note that we also used a reduced ATP-sensitive version of ATeam^RK^-p92 (bottom panel) to demonstrate that the FRET signal is due to ATP-sensing. Scale bars represent 5 μm. Each experiment was repeated three-four times.

To estimate the ATP level within the tombusviral replication compartment, we used a FRET-based biosensor [54], which was previously adapted to estimate ATP levels [32,33]. Briefly, ATeam- p92^pol^ can measure ATP level due to the conformational change in the enhanced ε subunit of the bacterial F_0_F_1_-ATP synthase upon ATP binding [32,33]. The ε subunit bound to ATP draws the CFP and YFP fluorescent tags in close vicinity, increasing the FRET signal in confocal laser microscopy (Fig 11A). On the contrary, the ε subunit in the ATP-free form is present in an extended conformation, which places CFP and YFP tags in a distal position, thus reducing the FRET signal (Fig 11A) [54]. We found previously [32,33] that the ATeam-tagged p92^pol^ is a fully functional RdRp, which localizes to the viral replication compartment representing aggregated peroxisomes. Since these experiments are best performed in the presence of glucose in yeast media [33], we used pdc1Δ yeast strain expressing Pdc1p from a plasmid. We found that the ATP level was ~4-fold higher in pdc1Δ yeast strain expressing WT Pdc1p than in the control lacking *PDC1* or expressing Pdc1^S455F^ mutant (Fig 11B). Expression of the low-sensitive variant of ATeam (ATeam^RK^-p92) [33,54] in pdc1Δ yeast strain expressing Pdc1p showed low FRET values, confirming that the FRET data is derived from the ATP biosensor in this assay. Overall, the obtained data support the model that Pdc1p is recruited into the tombusvirus replication compartment in yeast to facilitate the generation of ATP locally for viral RNA synthesis.

**Fig 12:**
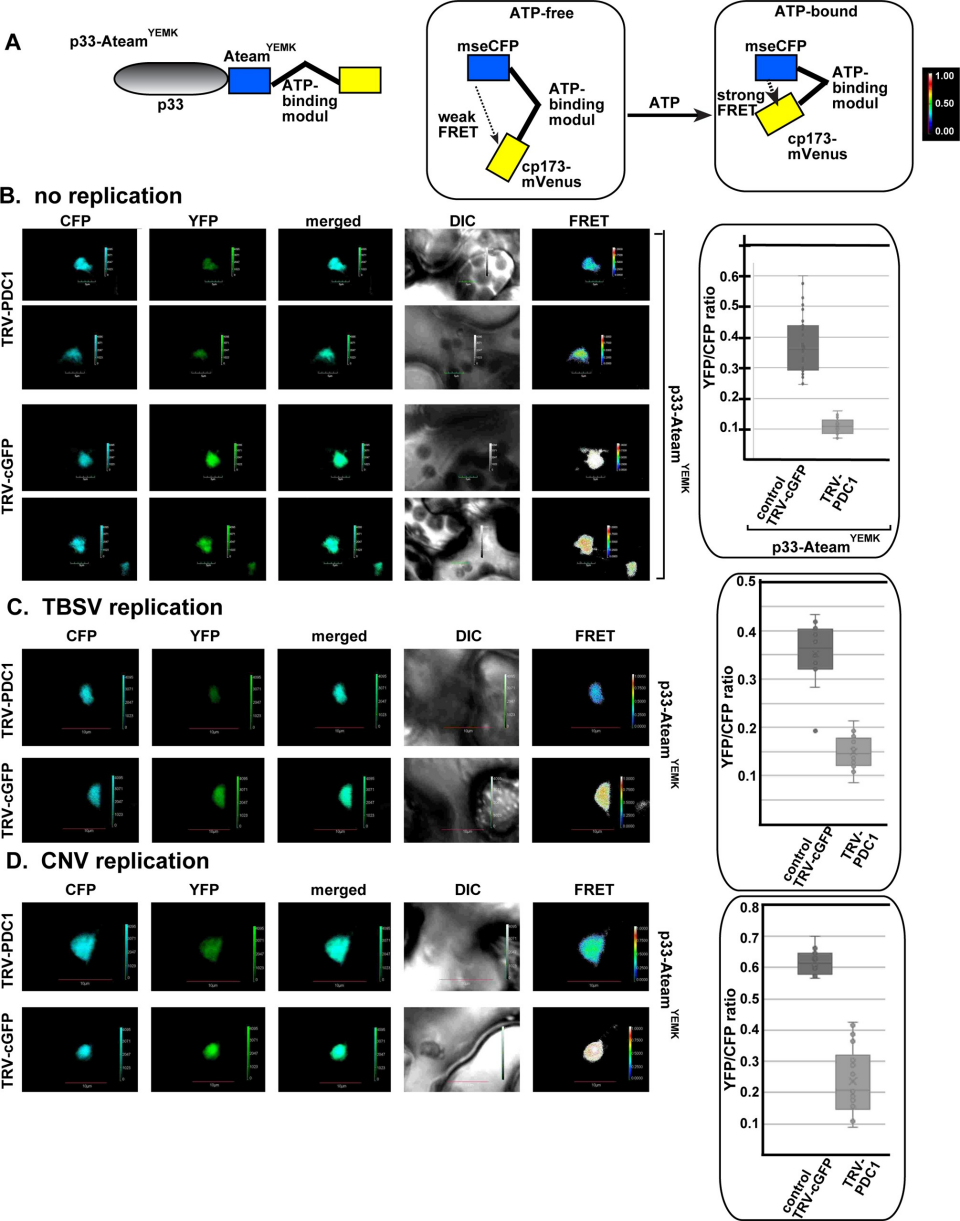
Knockdown of the cellular Pdc1 fermentation enzyme inhibits ATP accumulation within the tombusvirus replication compartment in *N*. *benthamiana*. (A) A scheme of the FRET-based detection of cellular ATP within the replication compartment. The enhanced ATP biosensor, ATeam^YEMK^ was fused to TBSV p33 replication protein. (B) Knock-down of Pdc1 mRNA level by VIGS in *N*. *benthamiana* was done using a TRV vector. Twelve days latter, expression of p33-ATeam^YEMK^ was done in upper *N*. *benthamiana* leaves by agroinfiltration. The YFP signal was generated by mVenus in p33-ATeam^YEMK^ via FRET 1.5 days after agro-infiltration. The FRET signal ratio is shown in the right panels. The more intense FRET signals are white and red (between 0.5 to 1.0 ratio), whereas the low FRET signals (0.1 and below) are light blue and dark blue. We also show the average quantitative FRET values (obtained with ImageJ) for 10–20 samples on the graph. Note that *N*. *benthamiana* plants were mock-inoculated. (C-D) Comparable experiments with p33-ATeam^YEMK^ in the Pdc1 knockdown *N*. *benthamiana* plants infected with the peroxisomal TBSV and CNV tombusviruses. See further details in panel B.

**Fig 13:**
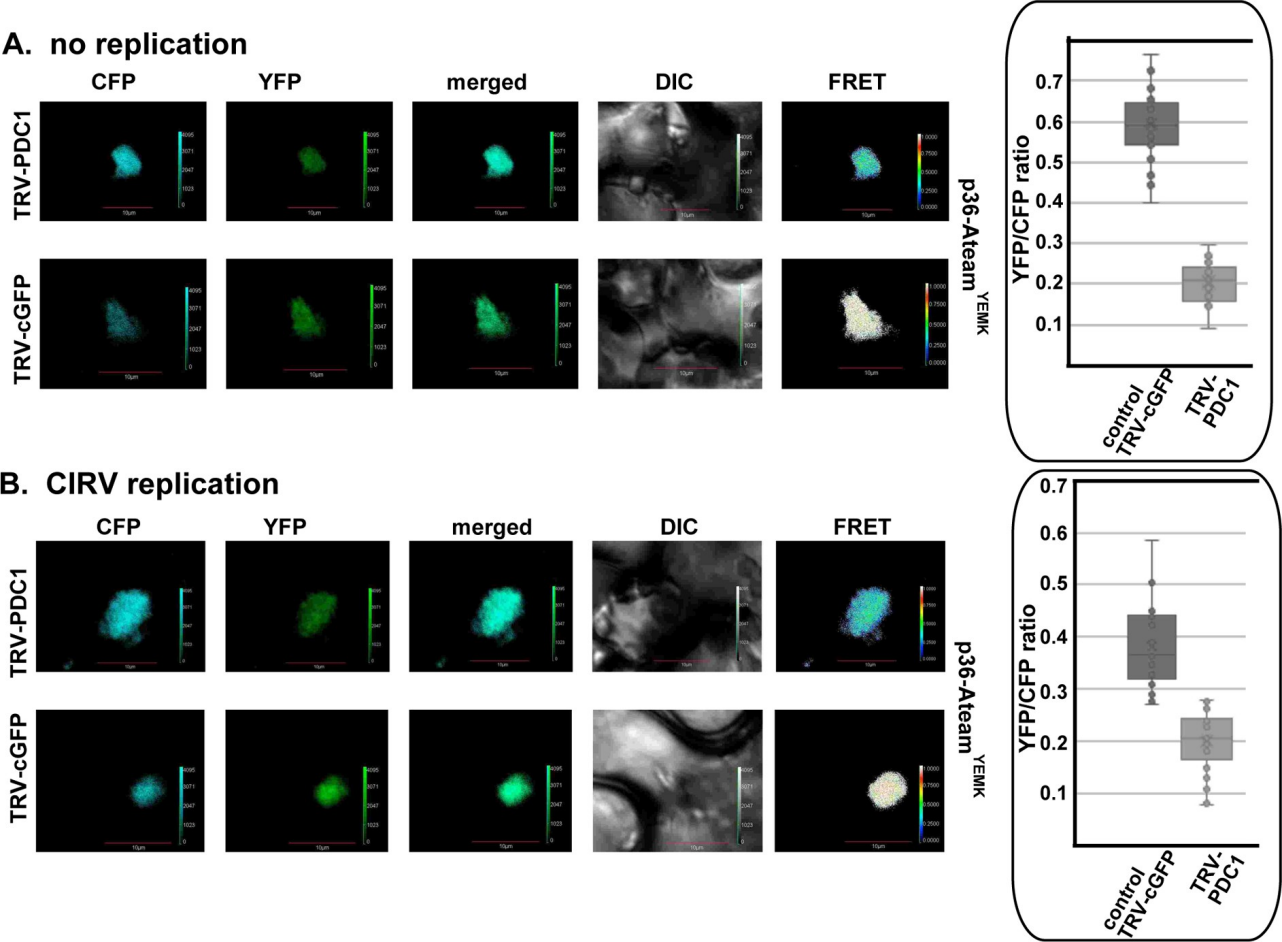
The co-opted cellular Pdc1 fermentation enzyme affects ATP accumulation within the CIRV replication compartment in *N*. *benthamiana*. (A) Knock-down of Pdc1 mRNA level by VIGS in *N*. *benthamiana* was done using a TRV vector. Twelve days later, expression of the CIRV p36-ATeam^YEMK^ was done in upper *N*. *benthamiana* leaves by agroinfiltration. The FRET-based confocal microscopy analysis was performed 1.5 days after agro-infiltration. The FRET signal ratio is shown in the right panels. We show the average quantitative FRET values for 10–20 samples on the graph. Note that *N*. *benthamiana* plants were mock-inoculated. (B) Comparable experiments to those in panel A, except CIRV supported repRNA replication in the cells. See further details in panel A.

To confirm that tombusviruses co-opt Pdc1 into the viral replication compartment to support efficient ATP generation in plants, we expressed p33-ATeam replication protein in *N*. *benthamiana* leaves, which were either silenced for Pdc1 expression or not (Fig 12A). The obtained data showed up to a ~4-fold reduction in ATP production within the viral replication compartment in the Pdc1 knockdown plants versus the control plants (Fig 12B). Similar experiments with *N*. *benthamiana* infected with TBSV showed a ~3-fold reduction in ATP level within the viral replication compartment in the knockdown plants versus the control plants (Fig 12C). Intensive TBSV replication likely uses up some of the produced ATP in the latter experiments as we observed previously [33]. Applying the same approach showed that similar pictures on ATP production within the viral replication compartment in the Pdc1-knockdown plants versus the control plants exist during the peroxisomal CNV (Fig 12D) and the mitochondrial CIRV (Fig 13) infections of *N*. *benthamiana* leaves. However, we did observe a range in ATP production [between ~3-fold (Fig 13A) and ~2-fold (S5 Fig)] in the absence of CIRV replication within the viral replication compartment in the Pdc1-knockdown plants versus the control plants in different experiments. It is possible that glucose concentrations and/or the efficiency of fermentation within the viral replication compartments in leaves are influenced by several physiological processes in plants. Nevertheless, the emerging picture is that subversion of Pdc1 into the viral replication compartment is required to support efficient ATP generation locally.

**Fig 14:**
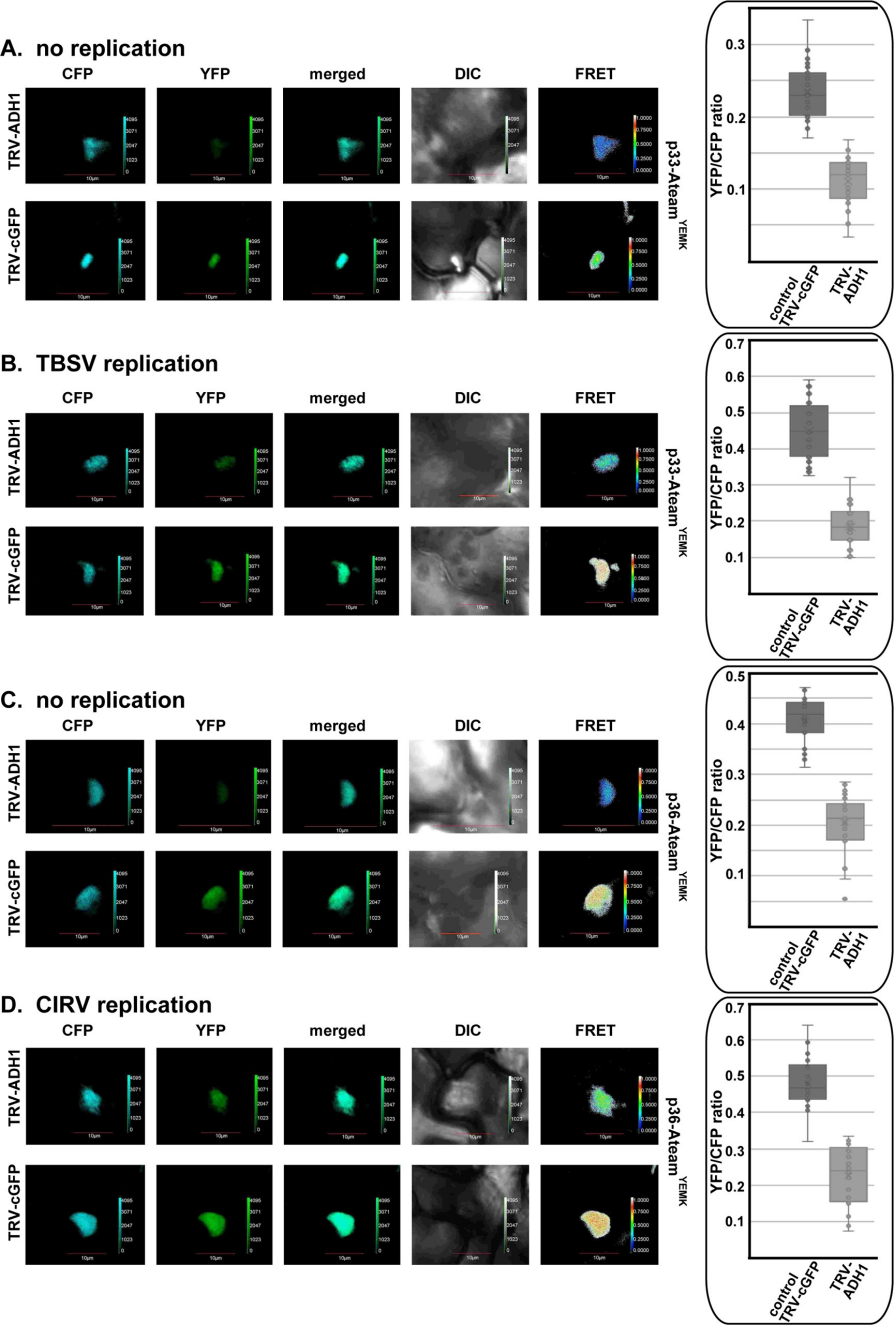
The co-opted cytosolic Adh1 is needed for ATP generation within the tombusvirus replication compartment in plants. (A-D) Knock-down of Adh1 mRNA level by VIGS in *N*. *benthamiana* was done using a TRV vector. Twelve days latter, expression of TBSV p33-ATeam^YEMK^ (panels A-B) or the CIRV p36-ATeam^YEMK^ (panels C-D) was done in upper *N*. *benthamiana* leaves by agroinfiltration. The YFP signal was generated via FRET 1.5 days after agro-infiltration. The FRET signal ratio is shown in the right panels. We show the average quantitative FRET values for 10–20 samples on the graph. Note that *N*. *benthamiana* plants were mock-inoculated or the plants supported TBSV and CIRV repRNA replication as shown. See further details in Fig 12B. Scale bars represent 10 μm. Each experiment was repeated three or four times.

Because Pdc1 works together with Adh1 in the fermentation pathway, we were curious if tombusviruses also co-opt Adh1 into the viral replication compartment to support efficient ATP generation locally in plants. Therefore, we expressed the p33-ATeam replication protein in *N*. *benthamiana* leaves, which were either silenced for Adh1 expression or not. We observed a ~2-fold reduction in ATP production within the viral replication compartment in the Adh1 knockdown plants versus the control plants (Fig 14A). Similar experiments with *N*. *benthamiana* infected with TBSV or CIRV also showed a ~2-fold reduction in ATP-level within the viral replication compartment in the knockdown plants versus the control plants (Fig 14). Based on these findings, we propose that subversion of both Pdc1 and Adh1 fermentation enzymes by tombusviruses facilitates the glycolytic process to produce plentiful ATP locally within the replication compartment in *N*. *benthamiana* leaves.

### Dependence of bamboo mosaic virus and tobacco mosaic virus replication on the fermentation pathway in plants

To investigate if additional plant viruses also depend on the fermentation pathway for robust replication, we chose bamboo mosaic virus (BaMV), a potexvirus, and tobacco mosaic virus (TMV), a tobamovirus, which are unrelated to TBSV.

**Fig 15:**
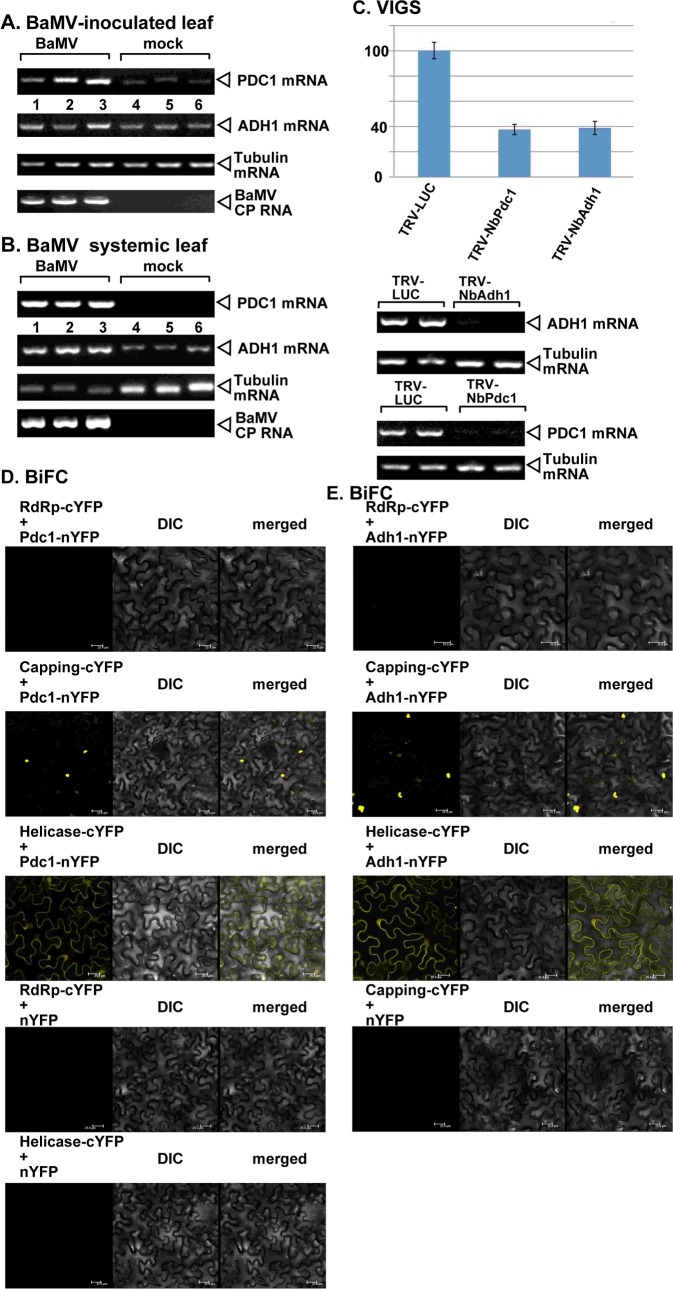
Dependence of BaMV replication on Pdc1 and Adh1 proteins in *N*. *benthamiana*. (A) Top panels: semi-quantitative RT-PCR analysis of NbPdc1 and NbAdh1 mRNA levels at 3 dpi in *N*. *benthamiana* leaves infected with bamboo mosaic virus (BaMV) or mock-inoculated. Third panel: RT-PCR analysis of tubulin mRNA level in the same plants. Bottom panel: RT-PCR detection of the BaMV CP subgenomic RNA. (B) Semi-quantitative RT-PCR analysis of NbPdc1 and NbAdh1 mRNA levels at 7 dpi in *N*. *benthamiana* leaves infected with BaMV or mock-inoculated. Third panel: RT-PCR analysis of tubulin mRNA level in the same plants. Bottom panel: RT-PCR detection of the BaMV CP subgenomic RNA. Each experiment was repeated three times. (C) Knockdown of Pdc1 or Adh1 mRNA levels inhibits BaMV replication in *N*. *benthamiana* plants. Top panel: Accumulation of the BaMV genomic (g)RNA in the Pdc1-silenced *N*. *benthamiana* plants 2.5 days post-inoculation (dpi) in the inoculated leaves was measured by quantitative RT-PCR. Inoculation of BaMV gRNA was done 12 days after silencing of Pdc1 or Adh1 expression. Agroinfiltration of tobacco rattle virus (TRV) vector carrying NbPdc1 or NbAdh1 or luciferase (LUC, as a control) sequences was used to induce VIGS. Second panel: RT-PCR analysis of tubulin mRNA level in the silenced and control plants. Each experiment was repeated three times. (D-E) The BaMV ORF1-capping-cYFP or ORF1-helicase-cYFP or ORF1-RdRp-cYFP domains of the replicase protein and the AtPdc1-nYFP (panel D) or AtAdh1-nYFP (panel E) proteins were expressed via agroinfiltration. The DIC and the merged images are also shown. The BiFC signals were detected via confocal microscopy 2 days after agroinfiltration to *N*. *benthamiana* plants. Scale bars represent 25 μm.

We found that BaMV and TMV replication led to the efficient induction of both Pdc1 mRNA and Adh1 mRNA expression in the inoculated as well as the systemically-infected *N*. *benthamiana* leaves (Fig 15A and 15B and S6A and S6B Fig). VIGS-based silencing of Pdc1 level in *N*. *benthamiana* leaves resulted in a ~60% reduction in the accumulation of both BaMV and TMV gRNAs (Fig 15C and S6C Fig). Similarly, knocking down Adh1 level in *N*. *benthamiana* leaves reduced the accumulation of BaMV and TMV gRNAs by 70% and 60%, respectively (Fig 15C and S6C Fig). To test if BaMV can recruit the fermentation proteins directly through protein-protein interactions, we used a BiFC approach. Co-expression of either the capping enzyme domain or the helicase domain of the BaMV replicase with Pdc1 in *N*. *benthamiana* leaves resulted in punctate and cytosolic signals, respectively (Fig 15D). In contrast, co-expression of the RdRp domain of the BaMV replicase with Pdc1 did not produce signals, suggesting the lack of interaction. Interestingly, we also observed interaction between the capping enzyme domain or the helicase domain, but not the RdRp domain of the BaMV replicase with Adh1 in *N*. *benthamiana* leaves (Fig 15E). Therefore, it is possible that BaMV also exploits the fermentation pathway via interaction between the viral replicase and the fermentation enzymes. Based on these observations, we suggest that similar to tombusviruses, other unrelated and rapidly replicating plant viruses also depend on the fermentation pathway in plants. Further experiments will be needed on the mechanistic details on the role of the fermentation pathway in the replication of BaMV and TMV.

## Discussion

### Compartmentalization of the co-opted fermentation pathway in the tombusviral replication compartment to support tombusvirus replication

The viral replication proteins orchestrate the biogenesis of the large tombusviral replication compartment, including the numerous spherules/VRCs, which represent the sites of viral RNA replication [22,31,55]. The formation and operation of these virus-driven structures require subversion of numerous cellular proteins, membrane deformation, membrane proliferation, changes in lipid composition of the hijacked cellular membranes and intensive viral RNA synthesis. To obtain the necessary resources from the infected cells, tombusviruses have to rewire cellular pathways to fuel the biogenesis of the replication compartment. These robust processes require plentiful ATPs and molecular building blocks produced at the sites of replication or delivered there. The emerging picture with tombusviruses is that by co-opting the aerobic glycolysis, the ATP molecules are produced and utilized within the replication compartment [32,33]. However, the aerobic glycolysis requires the replenishing of the NAD^+^ pool, which is used by the glycolytic GAPDH to produce NADH. NAD^+^ is efficiently generated by the fermentation pathway, which also utilizes pyruvate, the end product of the glycolytic pathway [36,38]. Accordingly, in the current work we show the efficient recruitment of Pdc1 and Adh1 fermentation enzymes into the viral replication compartment. Depletion of Pdc1 combined with deletion of the homologous *PDC5* in yeast or knockdown of Pdc1 and Adh1 in plants reduced the efficiency of tombusvirus replication. A complementation approach revealed that the enzymatically functional Pdc1p is required to support tombusvirus replication. We provide evidence that both Pdc1 and Adh1 enzymes are required for efficient generation of ATP within the replication compartment based on the measurements with an ATP biosensor inside the viral replication compartment (Figs 11–14). Moreover, in vitro works show the pro-viral function of Pdc1 during the assembly of the viral replicase and the activation of the p92 RdRp, both of which require the co-opted ATP-driven Hsp70 protein chaperone.

Is the co-opted fermentation pathway only required for facilitating ATP production within the tombusviral replication compartment? Albeit not studied in this work, it is very likely that the co-opted aerobic glycolysis in combination with the subverted fermentation pathway also provide plentiful metabolic precursors, which could be utilized by the cell to make molecular building blocks, such as ribonucleotides, lipids and amino acids [36,37]. These newly made molecular building blocks are likely exploited by tombusviruses to build the viral replication compartment and support intensive viral RNA synthesis. Accordingly, high glucose concentration stimulates TBSV replication in yeast, whereas blocking the aerobic glycolysis with 2DG compound strongly inhibited TBSV accumulation in yeast and plants [56].

Why are the relatively inefficient aerobic glycolytic and fermentation pathways co-opted by tombusviruses? These metabolic pathways are present in the cytosol, thus easily accessible for subversion by the cytosolic tombusviruses. Moreover, the ATP generation by the aerobic glycolytic and fermentation pathways is fast if plentiful glucose is present in the cells. Plants produce plentiful glucose based on chloroplasts, thus glucose is not expected to be rate limiting for the aerobic glycolytic and fermentation pathways in the infected plant cells. Moreover, these metabolic pathways do not require free oxygen, which could be an advantage for tombusviruses that also replicate efficiently in plant roots. Moreover, tombusviruses require the synthesis of new phospholipids and ribonucleotides [26,57]. The nexus point of the metabolic pathways, which is pyruvate, the end-product of glycolysis, has to be re-routed into the fast fermentation pathway. This then leads to the rapid regeneration of NAD^+^ to replenish the glycolytic pathway. NAD^+^ is also necessary for the biosynthesis of nucleotides and amino acids, and the fermentation pathway supports fast glucose flux through glycolysis. Thus, the rapid regeneration of NAD^+^ allows fast incorporation of glucose into metabolites [36–38]. Altogether, by providing plentiful precursor compounds in the cytosol, the aerobic glycolytic and fermentation pathways are far more efficient to facilitate the production of molecular building blocks than the oxidative phosphorylation pathway [36–38]. Then, the generated new metabolites can be exploited by tombusviruses to build extensive replication.

Why is compartmentalization of the aerobic glycolytic and fermentation pathways in the replication compartment advantageous for tombusviruses? The combined subversion of the aerobic glycolytic and fermentation pathways allows for the rapid production of ATP locally, including replenishing of the regulatory NAD^+^ pool by the fermentation pathway. Then, the locally produced ATP could be used efficiently by the co-opted ATP-dependent host factors, such as the Hsp70 protein chaperone, the ESCRT-associated Vps4 AAA ATPase and the pro-viral DEAD-box helicases [32,33]. These co-opted host factors are required for pro-viral processes, including VRC assembly, the activation of p92 RdRp, and the utilization of both ssRNA templates and dsRNA replication intermediates for viral RNA synthesis [22,32,33]. By producing the ATP locally within the replication compartment, tombusviruses do not need to compete with cellular processes for the common ATP pool and all the molecular processes could be accelerated by the high local concentration of ATP within the replication compartment. It is also possible that the feedback regulation of these metabolic processes by the cell is less efficient when compartmentalized in the viral replication compartment. Overall, there is an evolutionary pressure for tombusviruses to replicate fast and speed ahead of antiviral responses of the hosts and to outcompete other pathogenic viruses. Therefore, there are numerous advantages for tombusviruses to subvert the cellular aerobic glycolytic and fermentation pathways to support the infection process.

Aerobic glycolysis is induced during cancer and other diseases as well, including type 2 diabetes, amyloid-based brain diseases, wound repair and oncogenic virus infections [58–61]. Switching to the aerobic glycolytic metabolism can also occur with healthy cells, for example, during Endothelial cell differentiation, monocytes-based trained immunity, in rapidly dividing cells during embryogenesis, during T cell differentiation and motor adaptation learning in human brain [37,58,62,63]. The fetal heart primarily produces ATP via glycolytic metabolism [38]. All these cells/tissues utilize aerobic glycolysis as a metabolic compromise to provide ATP and produce enough new metabolic compounds to perform their functions.

In summary, we show evidence that TBSV exploit the fermentation pathway to support rapid virus replication. The dependence on the fermentation pathway is also shown for several other related and unrelated plant viruses. These viruses induce the fermentation pathway, thus indicating that a broad range of viruses takes advantage of the rapid cytosolic generation of ATP and numerous metabolic precursors. It will also be interesting to learn if other (+)RNA viruses exploit the aerobic glycolytic and fermentation pathways for their replication. Because all plant, animal and human (+)RNA viruses require the biogenesis of the membranous viral replication compartment/organelle, thus they likely use plenty ATP and they might depend on the production of new metabolic precursors, it is possible that hijacking the aerobic glycolytic and fermentation pathways occurs in other virus-host interactions as well. This could open up new common antiviral strategies targeting the fermentation pathway.

## Materials and methods

### Plant materials, yeast strain and plasmids

Wild type *N*. *benthamiana* plants were potted in soil and placed in growth room at 25°C under a 16-h-light/8-h-dark cycle. *S*. *cerevisiae* strain BY4741 (MATa his3Δ1 leu2Δ0 met15Δ0 ura3Δ0) was purchased from Open Biosystems. Yeast strains pdc1Δ was from the YKO library (Openbiosystems). To create pdc5Δ yeast strain, the hygromycin resistance gene hphNTI was PCR-amplified from vector pFA6a–hphNT1 (Euroscarf) [64] with primers #7504 and primers #7505 and the PCR product was transformed into BY4741. To generate GAL1::PDC1 pdc5Δ and GAL1::HA-PDC2 yeast strains, the transformants with GAL1 promoter along with the nourseothricin resistance gene were PCR-amplified from plasmid pYM-N23 with primers #7508 and #7509 or from pYM-N24 with #7475 and #7476 and then transformed into pdc5Δ and BY4741 yeast strains, respectively. Yeast strain NMY51 was obtained from Dualsystems. Plasmids and their constructions are listed in S1 and S2 Tables and primers used are described in S3 Table.

### Analysis of virus replication in yeast

To determine the effect of Pdc1 on the replication of TBSV in yeast, BY4741, pdc5Δ and GAL1::PDC1 pdc5Δ strains were transformed with HpGBK-CUP1-Flagp33, LpGAD-CUP1-Flag92 and UpCM189-Tet-DI72. TBSV replication was induced by growing cells at 23°C in SC-ULH^−^ (synthetic complete medium without uracil, leucine and histidine) medium supplemented with 2% galactose or 2% raffinose for 16 h. Then, yeast cultures were resuspended in SC-ULH^−^ medium supplemented with 50 μM CuSO_4_ and 2% galactose or 2% raffinose, and grown for 24 h at 23°C.

To complement TBSV or CIRV replication with Pdc1 in pdc1Δ yeast strain, plasmids HpGBK-CUP1-Hisp33/Gal-DI72 and LpGAD-CUP1-Hisp92 or HpESC-CUP1-Flagp36/Gal-DI72 and LpESC-CUP1-Flagp95, respectively, were co-transformed with UpCM189-Tet-empty or UpCM189-Tet-HisPdc1 or UpCM189-Tet-Pdc1 into yeast strain. To test if the enzymatic function of Pdc1 is required for TBSV replication, plasmids HpGBK-CUP1-Hisp33/Gal-DI72, LpGAD-CUP1-Hisp92 and UpCM189-Tet-Pdc1^S455F^ were co-transformed into pdc1Δ yeast strain. Transformed yeast cells were pre-grown in 2 ml SC-ULH^−^ medium supplemented with 2% galactose and 100 μM BCS for 16 h at 23°C. Then, yeast cultures were resuspended in SC-ULH^−^ medium supplemented with 2% galactose and 50 μM CuSO_4_ and grown for 24 h at 23°C.

### Co-purification assay

To understand the dynamics of Pdc1 association with the viral replicase, transformed yeast cells were pre-grown in SC-ULH^−^ medium supplemented with 2% glucose and 100 μM BCS at 29°C for 16 h. Then yeast cultures were centrifuged and the pellets were resuspended in SC-ULH^−^ medium supplemented with 2% galactose and 100 μM BCS and grown at 23°C for 24 h, followed by culturing yeast cells in SC-ULH^−^ medium supplemented with 2% galactose and 50 μM CuSO_4_ at 23°C for 6 h. Next, the yeast cells were shifted to SC-ULH^−^ medium supplemented with 2% glucose and cycloheximide (100 μg/ml) and samples were taken at 0, 1 h and 2.5 h time points. Yeast cultures were treated with formaldehyde and glycine and performed Flag-immunoaffinity purification as described below.

Co-purification assay from plants was performed by slight modifications of a previously described method [65]. Briefly, *N*. *benthamiana* leaves were co-infiltrated with agrobacterium carrying pGD-HA-AtPdc1, pGD-GFP-HA, pGD-T33-Flag, pGD-p19 and pGD-empty. Then, samples were harvested at 2.5 days post agroinfiltration and ground in cooled mortar in PPEB buffer (10% [v/v] glycerol, 25 mM Tris-HCl, pH 7.5, 1 mM EDTA, 150 mM NaCl, 10 mM DTT, 0.5% [v/v] Triton X-100 and protease inhibitor cocktail). The supernatant was incubated with anti-FLAG M2 affinity agarose (Sigma-Aldrich) using Bio-spin chromatography columns (Bio-rad) for 2 h at 4°C on a rotator, followed by washing with the CP buffer (10% [v/v] glycerol, 25 mM Tris-HCl, pH 7.5, 1 mM EDTA, 150 mM NaCl, 1mM DTT and 0.1% [v/v] Triton X-100). Elutions of the purified proteins were as described in the co-purification assay in yeast [66].

### Knockdown of NbPdc1 and NbAdh1 in *N*. *benthamiana* plants by VIGS

The VIGS-based knockdown of host genes in *N*. *benthamiana* was performed as described previously [65]. To generate VIGS constructs TRV2-NbPdc1 and TRV2-NbAdh1, cDNA fragments were PCR-amplified with primers #5847/#5848 and #7911/#7912 from *N*. *benthamiana* cDNA preparations and inserted into the plasmid pTRV2 [67]. At 12 days after VIGS treatment of *N*. *benthamiana* (pTRV1 together with pTRV2-NbPdc1 or pTRV2-NbAdh1 or pTRV2-cGFP or TRV-LUC), the levels of *N*. *benthamiana* NbPdc1 and NbAdh1 mRNAs were determined by semi-quantitative RT-PCR. Then, the silenced leaves were either sap inoculated with TBSV, CIRV or TCV inocula or agroinfiltrated with pGD-CNV^20KSTOP^ or pGD-CIRV, to launch virus replication. At different time points, samples from the inoculated and systemically-infected leaves were collected, followed by total RNA extraction and northern blot analysis as described previously [65]. In case of BaMV, the VIGS-silenced leaves were sap-inoculated with BaMV inocula to launch viral replication. Then, the inoculated leaves were collected at 2.5 dpi, followed by total RNA extraction and quantitative real-time RT-PCR analysis as described previously [68]. The *N*. *benthamiana* EF1α gene was used as an internal control to normalize the level of viral gene expression.

The VIGS-silenced NbPdc1 and NbAdh1 leaves of *N*. *benthamiana* were agroinfiltrated with pJL-36 vector carrying TMV cDNA and plant samples were collected 2 days after infection from the inoculated leaves. The TMV RNA levels were measured by northern blot analysis.

### Plant protoplasts preparation and viral RNA transfection

Protoplasts preparation from plant leaves was performed by some modifications of a previously described method [69]. Briefly, the NbPdc1-silenced or the mock-treated leaves were harvested at 12 days post VIGS silencing. Then, the leaves were sliced into 0.5–1 mm strips, digested with an enzyme solution containing 1.2% [w/v] Cellulase, 0.16% [w/v] Macerozyme, 0.12% [w/v] BSA and 0.5 M mannitol. To improve the isolation of protoplasts, leaf strips were vacuum infiltrated for 20 min in the dark using Vacufuge Plus (Eppendorf) and further digested in the dark for at least 3 h at room temperature. The protoplasts preparations were passed through a sieve set (Scienceware Mini-Sieve Microsieve Set from Fisher cat# 14-306A) and collected by centrifugation at 900 rpm for 2 min, followed by washing once with the W5 solution (154 mM NaCl, 125 mM CaCl_2_, 5 mM KCl, 2 mM MES pH5.7) and re-suspending in the W5 solution. Then, 0.6 M sucrose was layered under the W5 solution with protoplasts and centrifuged at 900 rpm for 3 min. Protoplasts were transferred from the interface between the W5 solution and 0.6 M sucrose layers in the same amount of the W5 solution, followed by washing once with the W5 solution and re-suspending at 2 x10^5^ ml^–1^ in the MMG solution (4 mM MES pH5.7, 0.4 M mannitol and 15 mM MgCl_2_). For RNA transfection, protoplasts were incubated with the PEG-calcium transfection solution containing 40% PEG 4000, 0.2 M mannitol and 100 mM CaCl_2_ and either viral RNA transcripts or total RNA extracts obtained from virus-infected plants at room temperature for up to 15 min. The transfection mixtures were diluted with the W5 solution and centrifuged at 100 g for 2 min at room temperature and incubated in the WI solution (4 mM MES, pH 5.7, 0.5 M mannitol and 20 mM KCl). The protoplasts were harvested at 16 h or 24 h post-transfection and subjected to RNA extraction and northern blot analysis as mentioned above.

### Visualization and measurement of ATP levels in yeast and plants

To visualize ATP production within the TBSV replication compartments in yeast, the previously adapted ATeam-based biosensor LpGAD-ADH-ATeam^YEMK^-p92 (high sensitivity) and LpGAD-ADH-ATeam^RK^-p92 (low sensitivity) were utilized [33,54]. pdc1Δ yeast strain was co-transformed with HpGBK-CUP1-Hisp33 and UpCM189-Tet-HisPdc1 or UpCM189-Tet-HisPdc1^S455F^ or UpCM189-Tet. The transformed yeast cells were pre-grown in 2 ml SC-ULH^−^ medium supplemented with 2% glucose and 100 μM BCS for 16 h at 23°C. Then, the yeast cultures were re-suspended in SC-ULH^−^ medium supplemented with 2% glucose and 50 μM CuSO_4_ and grown for 3 h at 23°C. Then samples were collected for confocal laser microscopy analysis. FRET values (YFP/CFP ratio) were obtained based on the quantification of CFP and Venus images using ImageJ software and calculation using Microsoft Excel as described [33].

To measure the ATP level within the tombusvirus replication compartments in the NbPdc1- or the NbAdh1-silenced *N*. *benthamiana* leaves, the previously adapted ATeam-based biosensor pGD-p33-ATeam^YEMK^ or pGD-p36-ATeam^YEMK^ [33] were transformed into agrobacterium strain C58C1. In case of TBSV, the silenced or the control leaves of *N*. *benthamiana* (at 12 days after VIGS treatment using pTRV1 in combination with pTRV2-NbPdc1 or pTRV2-NbAdh1 or pTRV2-cGFP control) were co-agroinfiltrated with plasmids pGD-p33-ATeam^YEMK^ with or without pGD-p92 and pGD-DI72. In case of CNV, the silenced or the control leaves were co-agroinfiltrated with plasmids pGD-p33-ATeam^YEMK^ with or without pGD-CNV^20KSTOP^. In case of CIRV, the silenced or control leaves were co-agroinfiltrated with plasmids pGD-p36-ATeam^YEMK^ with or without pGD-p95 and pGD-DI72. Then samples were harvested at 1.5 day after agroinfiltration for the confocal microscopy analysis. FRET values (YFP/CFP ratio) were obtained based on the quantification of CFP and Venus images using ImageJ software and calculation using Microsoft Excel [33].

### Yeast cell free extract (CFE)-based in vitro replication assay

CFEs from BY4741, pdc5Δ and GAL1::PDC1 pdc5Δ yeast strains were prepared as described earlier [53,70]. These yeast stains were pre-grown in YPD or YPG media at 29°C for 16 h. Then, the yeast cultures were diluted (to 0.4 OD_600_) with fresh YPD or YPG media and grown at 29°C for 5 h, followed by 37°C treatment for 30 min. The individual CFE preparations were made following the published protocol [53,70] and adjusted to contain comparable amounts of total proteins. The in vitro CFE assay was performed in 20 μl total volume containing 2 μl of adjusted CFE, 0.5 μg DI-72 (+)RNA transcripts, 0.5 μg affinity-purified MBP-p33, 0.5 μg affinity-purified MBP-p92 (both recombinant proteins were obtained from E. coli) [71,72], 30 mM HEPES-KOH, pH 7.4, 150 mM potassium acetate, 5 mM magnesium acetate, 0.13 M sorbitol, 0.2 μl actinomycin D (5 mg/ml), 2 μl of 150 mM creatine phosphate, 0.2 μl of 10 mg/ml creatine kinase, 0.2 μl of RNase inhibitor, 0.2 μl of 1 M dithiothreitol (DTT), 2 μl of 10 mM ATP, CTP, and GTP and 0.1 mM UTP and 0.2 μl of ^32^P-UTP. Reaction mixtures were incubated for 3 h at 25°C, followed by phenol/chloroform extraction and isopropanol/ammonium acetate (10:1) precipitation. The ^32^P-UTP-labeled RNA products were analyzed in 5% acrylamide/8 M urea gels [53,70]. Additional methods used are described in S1 text.

## Supporting information

S1 Fig FHV replication depends on the expression of Pdc1/5 fermentation enzymes in yeast. (A) Depletion of Pdc1p in combination with deletion of the homologous *PDC5* inhibits FHV RNA replication in yeast. Top panels: northern blot analyses of FHV RNA1 and RNA3 using a 3’ end specific probe demonstrates reduced accumulation of FHV RNAs in GAL::PDC1 pdc5Δ yeast strain with depleted Pdc1p (raffinose-containing media) in comparison with the WT yeast strain or GAL::PDC1 pdc5Δ yeast strain with induced Pdc1p (galactose-containing media). Second panel: northern blot with 18S ribosomal RNA specific probe was used as a loading control. (B) The down-regulation of Pdc1 mRNA was confirmed with RT-PCR. Each experiment was repeated three times.
(PDF)

S2 Fig Pdc2 transcription factor, which regulates the expression of fermentation enzymes, is an essential host factor for tombusvirus replication in yeast. (A) Depletion of Pdc2p inhibits TBSV repRNA replication in yeast. Top panels: northern blot analyses of TBSV repRNA using a 3’ end specific probe demonstrates reduced accumulation of repRNA in GAL::PDC2 yeast strain with depleted Pdc2p (raffinose-containing media) in comparison with the WT yeast strain or GAL::PDC2 yeast strain with induced Pdc1p (galactose-containing media). Bottom image: western blot analysis of the level of HA-tagged Pdc2 protein with anti-HA antibody.
(PDF)

S3 Fig Additional experiments to show the recruitment of Pdc1 and Adh1 to the sites of tombusviral replication in *N*. *benthamiana*. Note that these experiments were performed under the same experimental conditions as shown in Fig 8. These images document that the RFP-MS2-CP sensor of the TBSV repRNA carrying the MS2 RNA hairpins is re-targeted to the sites of tombusvirus replication partially from the nucleus in *N*. *benthamiana* cells infected with CNV, likely due to the less robust replication of repRNAs in these cells when compared with those shown in Fig 8.
(PDF)

S4 Fig Negative control experiments for the BiFC studies. (A) See further details in Fig 9. (B-C) Western blot analysis of expression nYFP-AtPdc1 and nYFP-AtAdh1, respectively, in *N*. *benthamiana* with anti-Flag antibody.
(PDF)

S5 Fig The co-opted cellular Pdc1 fermentation enzyme affects ATP accumulation locally within the CIRV replication compartment in *N*. *benthamiana*. Knock-down of Pdc1 mRNA level by VIGS in *N*. *benthamiana* was done using a TRV vector as in Fig 13. Note that these experiments were performed under the same experimental conditions as shown in Fig 13A.
(PDF)

S6 Fig Dependence of TMV replication on Pdc1 and Adh1 proteins in *N*. *benthamiana*. (A) Top panels: semi-quantitative RT-PCR analysis of NbPdc1 and NbAdh1 mRNA levels at 2 dpi in *N*. *benthamiana* leaves infected with TMV or mock-inoculated. Third panel: RT-PCR analysis of tubulin mRNA level in the same plants. Bottom panel: Ribosomal RNA is shown as a loading control in an ethidium-bromide stained agarose gel. (B) Semi-quantitative RT-PCR analysis of NbPdc1 and NbAdh1 mRNA levels at 5 dpi in *N*. *benthamiana* leaves infected with TMV or mock-inoculated. Third panel: RT-PCR analysis of tubulin mRNA level in the same plants. Bottom panel: Ribosomal RNA is shown as a loading control in an ethidium-bromide stained agarose gel. (C) Knockdown of Pdc1 or Adh1 mRNA levels inhibits TMV replication in *N*. *benthamiana* plants. Top panel: Accumulation of the TMV genomic (g)RNA in the Adh1- or Pdc1-silenced *N*. *benthamiana* plants 2 dpi in the inoculated leaves was measured by northern blot. Inoculation of the TMV gRNA was done 12 days after silencing of Pdc1 or Adh1 expression. Agroinfiltration of the TRV-based vector carrying NbPdc1 or NbAdh1 or cGFP (as a control) sequences was used to induce VIGS. Second panel: RT-PCR analysis of tubulin mRNA level in the silenced and control plants. Each experiment was repeated three times. (D-E) Delayed development of TMV-induced symptoms is observed in the Adh1- or Pdc1-silenced *N*. *benthamiana* plants as compared with the control plants. Note the lack of phenotype in the Adh1- or Pdc1-silenced and mock-inoculated *N*. *benthamiana* plants. The pictures were taken at 8 dpi.
(PDF)

S1 Text Experimental procedures. (DOCX)

S1 Table Plasmids constructed in this study. (DOCX)

S2 Table Plasmids described in previous studies. (DOCX)

S3 Table Primers used in this study. (DOCX)
