# Supplementary figures and images for: Co-opting the fermentation pathway for tombusvirus replication: Compartmentalization of cellular metabolic pathways for rapid ATP generation
Source: PLoS Pathog. 2019 Oct 24;15(10):e1008092. doi: 10.1371/journal.ppat.1008092 (PMC6830812; doi:10.1371/journal.ppat.1008092)

S1 FIGURE

A. FHV

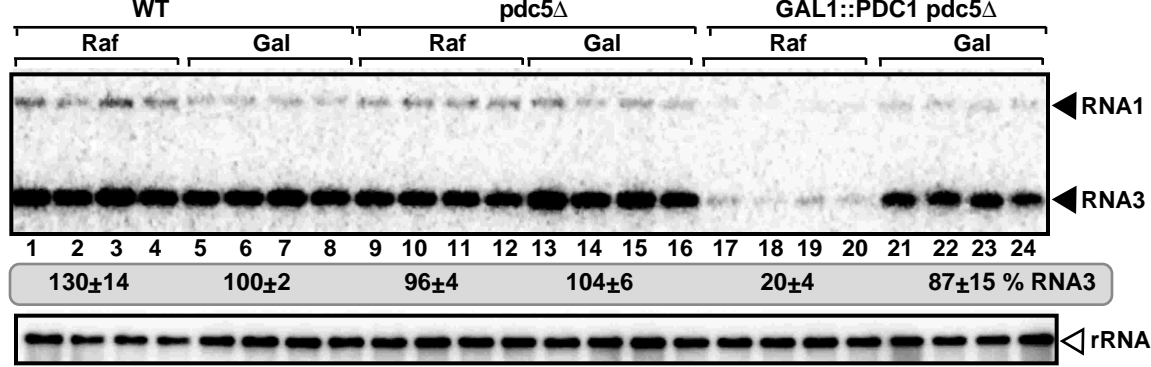

B

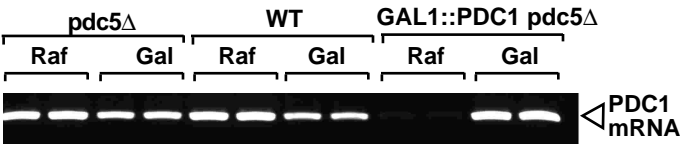

Supplement: S1 Fig — (A) Depletion of Pdc1p in combination with deletion of the homologous PDC5 inhibits FHV RNA replication in yeast. Top panels: northern blot analyses of FHV RNA1 and RNA3 using a 3’ end specific probe demonstrates reduced accumulation of FHV RNAs in GAL::PDC1 pdc5Δ yeast strain with depleted Pdc1p (raffinose-containing media) in comparison with the WT yeast strain or GAL::PDC1 pdc5Δ yeast strain with induced Pdc1p (galactose-containing media). Second panel: northern blot with 18S ribosomal RNA specific probe was used as a loading control. (B) The down-regulation of Pdc1 mRNA was confirmed with RT-PCR. Each experiment was repeated three times. (PDF) [file ppat.1008092.s001.pdf]

S2 FIGURE

TBSV

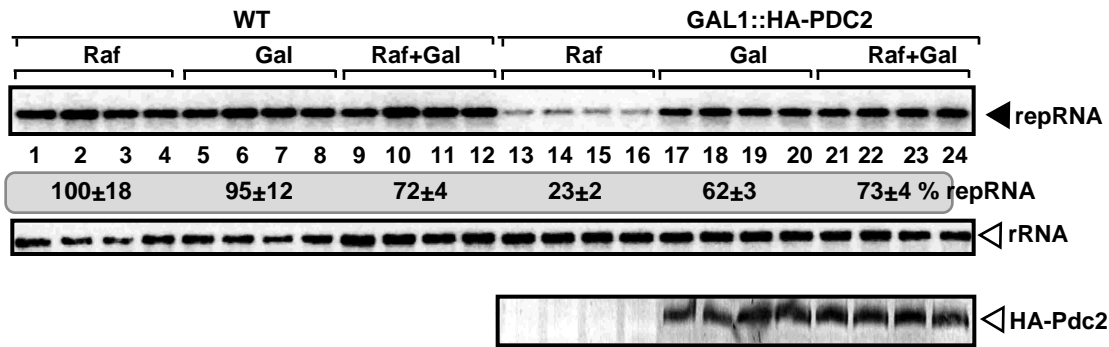

Supplement: S2 Fig — (A) Depletion of Pdc2p inhibits TBSV repRNA replication in yeast. Top panels: northern blot analyses of TBSV repRNA using a 3’ end specific probe demonstrates reduced accumulation of repRNA in GAL::PDC2 yeast strain with depleted Pdc2p (raffinose-containing media) in comparison with the WT yeast strain or GAL::PDC2 yeast strain with induced Pdc1p (galactose-containing media). Bottom image: western blot analysis of the level of HA-tagged Pdc2 protein with anti-HA antibody. (PDF) [file ppat.1008092.s002.pdf]

S3 FIGURE

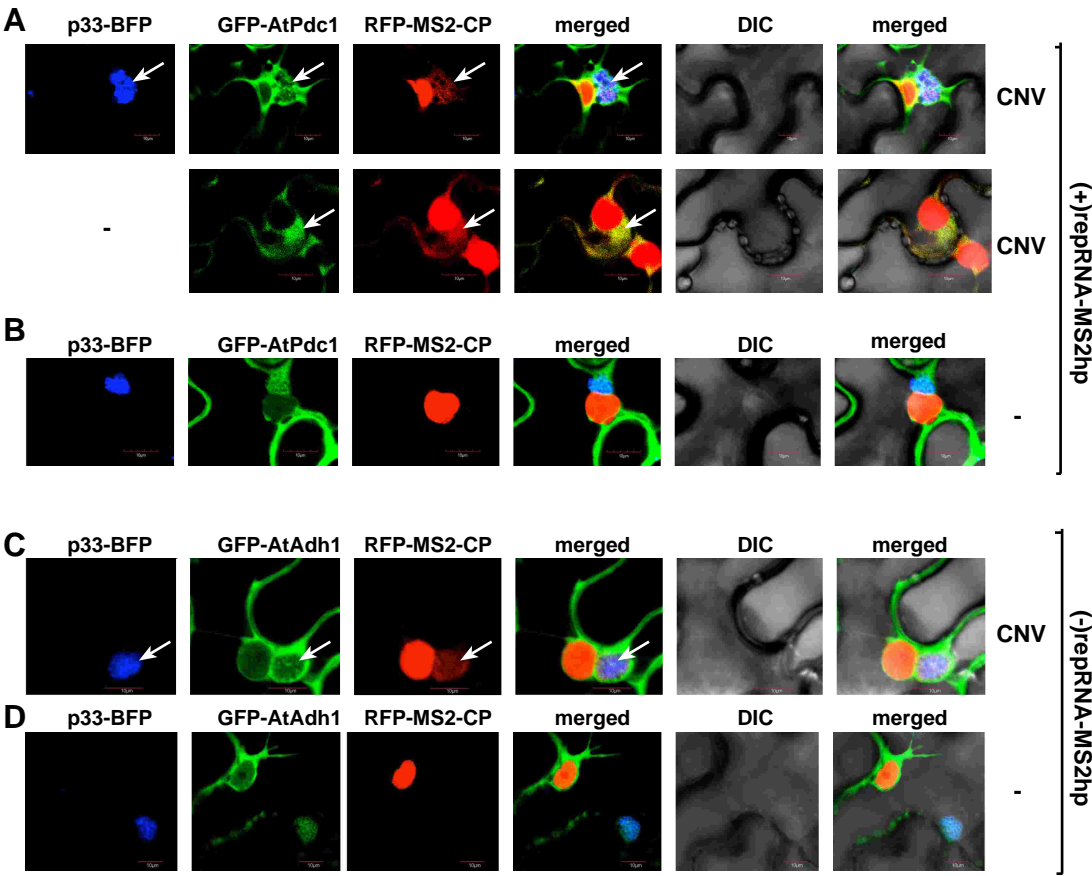

Supplement: S3 Fig — Note that these experiments were performed under the same experimental conditions as shown in Fig 8. These images document that the RFP-MS2-CP sensor of the TBSV repRNA carrying the MS2 RNA hairpins is re-targeted to the sites of tombusvirus replication partially from the nucleus in N. benthamiana cells infected with CNV, likely due to the less robust replication of repRNAs in these cells when compared with those shown in Fig 8. (PDF) [file ppat.1008092.s003.pdf]

## S4 FIGURE

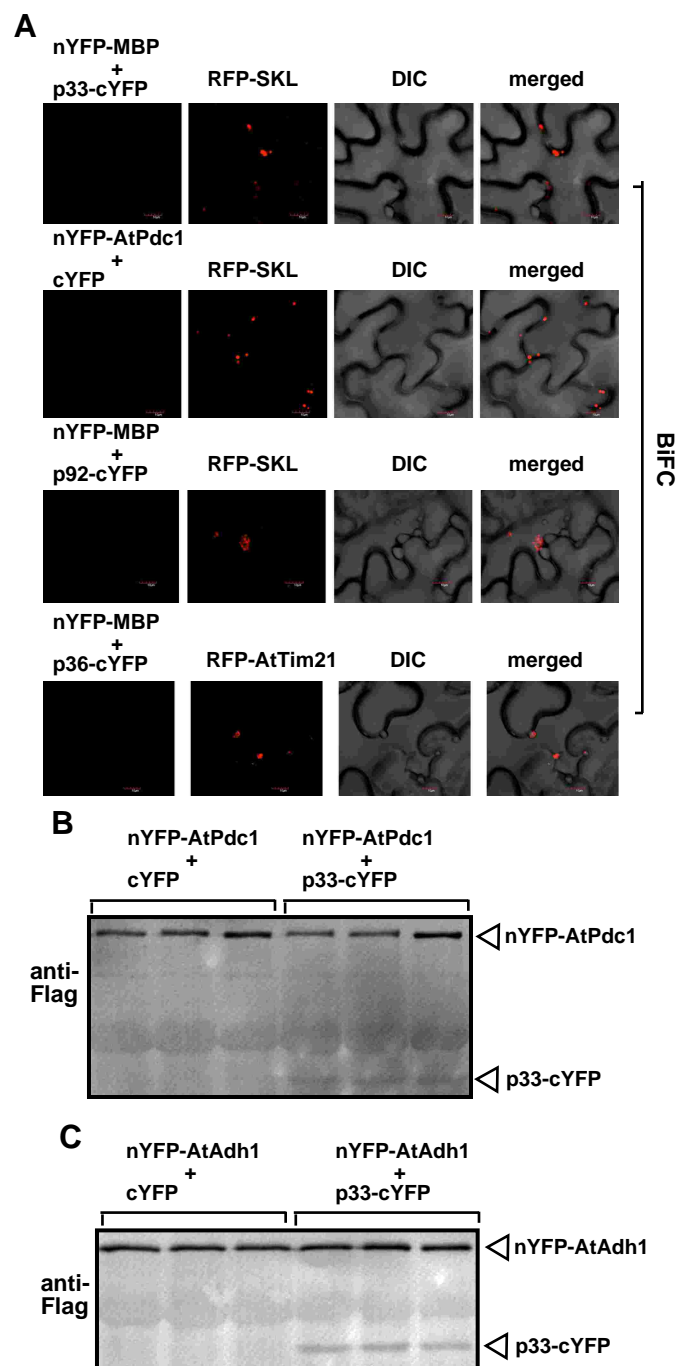

Supplement: S4 Fig — (A) See further details in Fig 9. (B-C) Western blot analysis of expression nYFP-AtPdc1 and nYFP-AtAdh1, respectively, in N. benthamiana with anti-Flag antibody. (PDF) [file ppat.1008092.s004.pdf]

## S5 FIGURE

**no replication**

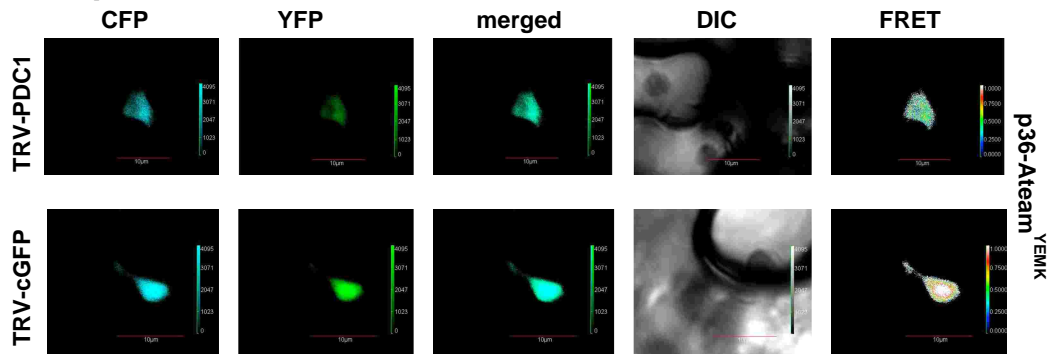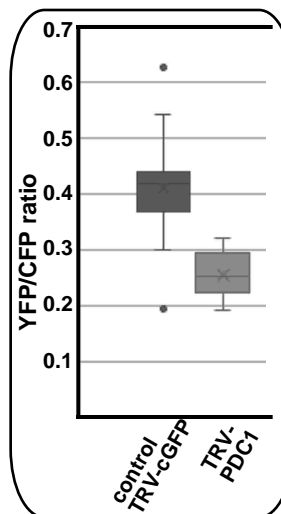

Supplement: S5 Fig — Knock-down of Pdc1 mRNA level by VIGS in N. benthamiana was done using a TRV vector as in Fig 13. Note that these experiments were performed under the same experimental conditions as shown in Fig 13A. (PDF) [file ppat.1008092.s005.pdf]

## S6 FIGURE

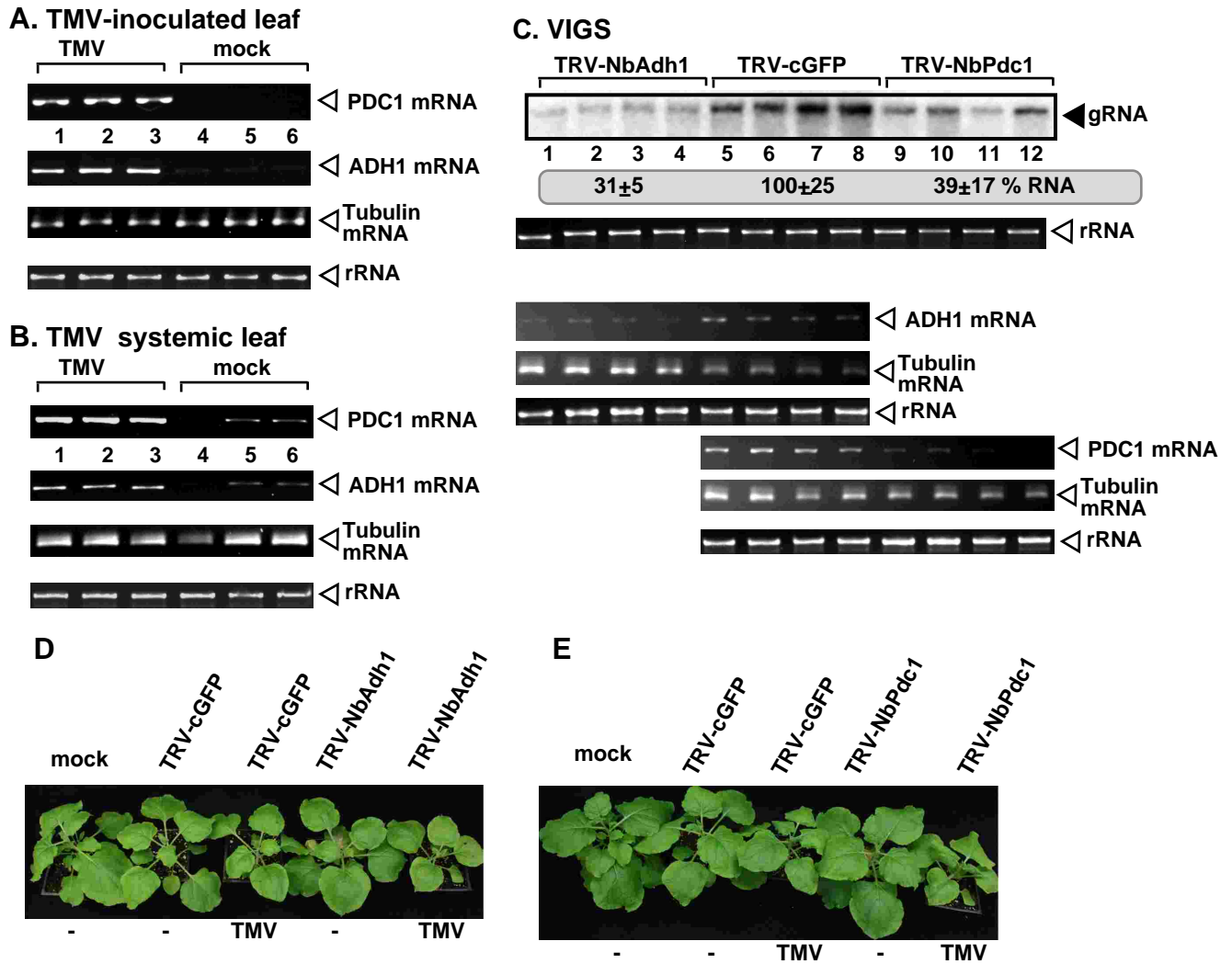

Supplement: S6 Fig — (A) Top panels: semi-quantitative RT-PCR analysis of NbPdc1 and NbAdh1 mRNA levels at 2 dpi in N. benthamiana leaves infected with TMV or mock-inoculated. Third panel: RT-PCR analysis of tubulin mRNA level in the same plants. Bottom panel: Ribosomal RNA is shown as a loading control in an ethidium-bromide stained agarose gel. (B) Semi-quantitative RT-PCR analysis of NbPdc1 and NbAdh1 mRNA levels at 5 dpi in N. benthamiana leaves infected with TMV or mock-inoculated. Third panel: RT-PCR analysis of tubulin mRNA level in the same plants. Bottom panel: Ribosomal RNA is shown as a loading control in an ethidium-bromide stained agarose gel. (C) Knockdown of Pdc1 or Adh1 mRNA levels inhibits TMV replication in N. benthamiana plants. Top panel: Accumulation of the TMV genomic (g)RNA in the Adh1- or Pdc1-silenced N. benthamiana plants 2 dpi in the inoculated leaves was measured by northern blot. Inoculation of the TMV gRNA was done 12 days after silencing of Pdc1 or Adh1 expression. Agroinfiltration of the TRV-based vector carrying NbPdc1 or NbAdh1 or cGFP (as a control) sequences was used to induce VIGS. Second panel: RT-PCR analysis of tubulin mRNA level in the silenced and control plants. Each experiment was repeated three times. (D-E) Delayed development of TMV-induced symptoms is observed in the Adh1- or Pdc1-silenced N. benthamiana plants as compared with the control plants. Note the lack of phenotype in the Adh1- or Pdc1-silenced and mock-inoculated N. benthamiana plants. The pictures were taken at 8 dpi. (PDF) [file ppat.1008092.s006.pdf]
