# Supplementary material for: Co-opting the fermentation pathway for tombusvirus replication: Compartmentalization of cellular metabolic pathways for rapid ATP generation
Source: PLoS Pathog. 2019 Oct 24;15(10):e1008092. doi: 10.1371/journal.ppat.1008092 (PMC6830812; doi:10.1371/journal.ppat.1008092)
Supplement: S1 Text — (DOCX) [file ppat.1008092.s007.docx]

**S1 Text. Experimental procedures.**

**Analysis of virus replication in yeast.** For testing FHV replication, yeast strain BY4741, pdc5∆ and GAL1::PDC1 pdc5∆ were co-transformed with pESC-His/CUP1/FHV/RNA1/Frameshift/TRSVR_RZ_, pGAD-Leu/CUP1/FHV/Protein-A/C-term/HA/FLAG and UpYES-NT empty vector. After pre-growing in 2 ml SC-ULH^−^ medium supplemented with 100 μM BCS and 2% galactose or 2% raffinose for 16 h at 23°C, yeast cells were resuspended in SC-ULH^−^ medium supplemented with 50 μM CuSO_4_ and 2% galactose or 2% raffinose and grown for 48 h at 23°C.

To study the effect of Pdc2 on tombusvirus replication, BY4741 and GAL1::HA-PDC2 yeast strains were transformed with HpGBK-CUP1-Hisp33/ADH-DI72 and LpGAD-CUP1-Hisp92. Transformed yeast cells were pre-grown in 2 ml SC-LH^−^ medium containing 100 μM BCS supplemented with 2% galactose or 2% raffinose or the combination of 2% galactose and 2% raffinose for 16 h at 23°C. Then yeast cultures were resuspended in SC-LH^−^ medium supplemented with 50 μM CuSO_4_ and 2% galactose or 2% raffinose or the combination of 2% galactose and 2% raffinose, and grown for 24 h at 23°C. All the replication assays in yeast were performed with standard RNA and protein extraction protocol as described previously [1,2].

**Yeast membrane two-hybrid assay.** The yeast membrane two-hybrid assay (MYTH) based on the split-ubiquitin system was performed as described previously [3]. Briefly, to determine the interaction between p33 and Pdc1 or Adh1-5 proteins, plasmids pGAD-BT2-N-Hisp33 was transformed with one of the following: pPR-N-RE, pPR-N-RE-Ssa1, pPR-N-RE-AtPdc1, pPR-N-RE-Pdc1, pPR-N-RE-AtAdh1, pPR-N-RE-Adh1, pPR-N-RE-Adh2, pPR-N-RE-Adh3, pPR-N-RE-Adh4 or pPR-N-RE-Adh5. Transformed yeasts colonies were suspended in 100 μl water, 8 μl of which cultured on TL^-^ plates as loading control or on TLHA^-^ plates to score protein interactions.

**Co-purification assay in yeast.** For co-purification of Pdc1 proteins with the TBSV p33/p92 replication proteins from yeasts, plasmids HpGBK-CUP1-Hisp33/Gal-DI72 and LpGAD-CUP1-Hisp92 (as a control) or HpGBK-CUP1-Flagp33/Gal-DI72 and LpGAD-CUP1-Flagp92 were co-transformed with UpYES-HisPdc1 or UpYES-HisAtPdc1 or UpCM189-Tet-HisPdc1 or UpCM189-Tet-HisPdc1^S455F^ into BY4741 yeast strain. For co-purification of Pdc1 proteins with the CIRV p36/p95 replication proteins from yeasts, plasmids HpGBK-CUP1-Hisp36/Gal-DI72 and LpGAD-CUP1-Hisp95 (as a control) or HpGBK-CUP1-Flagp36/Gal-DI72 and LpGAD-CUP1-Flagp95 were co-transformed with UpYES-HisPdc1or UpYES-HisAtPdc1 into BY4741 yeast strain.

For co-purification of Adh1-3 proteins with the TBSV p33/p92 replication proteins from yeasts, BY4741yeast strain was co-transformed with plasmids HpGBK-CUP1-Flagp33/Gal-DI72 and LpGAD-CUP1-Flagp92 or HpGBK-CUP1-Hisp33/Gal-DI72 and LpGAD-CUP1-Hisp92 (as a control) and one of the following: UpYES-HisAdh1, UpYES-HisAdh2, UpYES-HisAdh3 or UpYES-HisAtAdh1. For co-purification of Adh1 proteins with the CIRV p36/p95 replication proteins from yeasts, plasmids HpGBK-CUP1-Hisp36/Gal-DI72 and LpGAD-CUP1-His95 (as a control) or HpGBK-CUP1-Flagp36/Gal-DI72 and LpGAD-CUP1-Flag95 were co-transformed with UpYES-HisAdh1or UpYES-HisAtAdh1 into BY4741yeast strain.

All transformed yeast cells were pre-grown in SC-ULH^−^ media supplemented with 2% glucose and 100 μM BCS at 29°C for 16 h. Then yeast cultures were resuspended in SC-ULH^−^ medium supplemented with 2% galactose and 100 μM BCS and grown at 23°C for 24 h, followed by culturing yeast cells in SC-ULH^−^ medium supplemented with 2% galactose and 50 μM CuSO_4_ at 23°C for 6 h. The cultures were resuspended and incubated in 35 ml phosphate-buffered saline (PBS) buffer containing 1% formaldehyde for 1 h on ice to cross-link proteins. Then, glycine (adjusted to 0.1M) was added to quench the formaldehyde and the yeasts were incubated on ice for 5 min. Finally, yeast pellets were harvested after washing twice with PBS buffer and proteins were Flag-affinity purified as described previously [4].

**Pull-down assay.** The pull-down assay has been described [5]. Briefly, plasmids pGEX-His-RE-T33C, pGEX-His-RE-AtPdc1, pGEX-His-RE-AtAdh1 and pGEX-His-RE-Adh1 were transformed into Epicurion Bl21-codon-plus (DE3)-R1L cells (Stratagene) and the expression of those proteins were induced by IPTG. The *E. coli* cells were broken by sonication and the supernatants were used for purification with the help of a GST resin. For expression of MBP or MBP-tagged T33C, C36C, Pdc1 and AtPdc1, pMALc-2X, pMALc-2X-T33C, pMALc-2X-C36C, pMALc-2X-Pdc1 and pMALc-2X-AtPdc1, the expression plasmids were transformed into Epicurion Bl21-codon-plus (DE3)-R1L cells, followed by IPTG induction. After sonication, 100 μl lysates were incubated with 15 μl amylose resin (NEB) in Bio-spin chromatography columns for 1 h at 4°C, followed by four times washing with the column buffer [5]. The amylose columns containing the bound MBP proteins were then incubated with 1 μg of the purified GST-His_6_-tagged proteins for 2 h at 4°C. Then the washed beads were incubated in 1× SDS loading buffer for 10 min at 85°C. The MBP-tagged proteins were analyzed by SDS-PAGE electrophoresis followed by Coomassie blue-staining and GST-His_6_-tagged proteins were separated by SDS-PAGE for protein gel blot analysis with anti-His antibody.

**Confocal microscopy analysis of plant epidermal cells.** To analyze the subcellular localization of AtPdc1 or AtAdh1 in the presence or absence of viral components in *N. benthamiana* leaves, plasmids pGD-p33-BFP, pGD-p36-BFP, pGD-RFP-AtPdc1, pGD-p33-RFP, pGD-p36-RFP, pGD-BFP-AtAdh1, pGD-GFP-SKL (as a peroxisome matrix marker) and pGD-GFP-AtTim21 (as a mitochondrial marker) were transformed into C58C1 agrobacterium strain. Then agrobacterium suspensions with different combinations were infiltrated into *N. benthamiana* leaves, followed by tombusvirus inoculation with sap at 16 h post agroinfiltration. At 2.5 dpai, the agroinfiltrated leaves were subjected to confocal laser microscopy.

To detect interaction between AtPdc1/AtAdh1 with TBSV p33 or CIRV p36 replication proteins using bimolecular fluorescence complementation assay (BiFC), plasmids pGD-T33-cYFP, pGD-C36-cYFP, pGD-C-cYFP (as a negative control), pGD-nYFP-AtPdc1, pGD-nYFP-AtAdh1, pGD-nYFP-MBP (as a negative control), pGD-RFP-SKL (as a peroxisome marker) and pGD-RFP-AtTim21 (as a mitochondrial marker) were transformed into C58C1 agrobacterium strain. The agrobacterium transformants with different combinations were used to infiltrate *N. benthamiana* leaves, which were harvested and then subjected to confocal microscopy analysis at 2 days after agro-infiltration.

To demonstrate if AtPdc1and AtAdh1 were recruited into an active virus replication compartment, a modified TBSV repRNA consisting of six repeats of a hairpin RNA from MS2 bacteriophage, which binds to MS2 coat protein, was utilized [6]. *N. benthamiana* leaves were co-agroinfiltrated with different combinations of agrobacterium carrying pGD-p33-BFP (0.3 OD_600_), pGD-GFP-AtPdc1 (0.3 OD_600_), pGD-GFP-AtAdh1 (0.3 OD_600_), pGD-RFP-MS2-CP (0.5 OD_600_), pGD-p19 (0.3 OD_600_), pGD-(+)DI72-MS2hp (0.5 OD_600_), pGD-(-)DI72-MS2hp (0.5 OD_600_) and pGD-CNV^20KSTOP^ (0.4 OD_600_). Then, the samples were subjected to confocal microscopy analysis at 3.5 days post agro-infiltration.

To analyze interactions between AtPdc1 and AtAdh1 with the BaMV encoded replicase protein in plants, the transformed agrobacterium harboring either pEarleygate201-YN-AtPdc1 or pEarleygate201-YN-AtAdh1 was co-infiltrated into *N. benthamiana* leaves with one of the following agro-transformants: pEarleygate201-YC-BaMV-ORF1-capping, pEarleygate201-YC-BaMV-ORF1-Helicase, and pEarleygate201-YC-BaMV-ORF1-RdRp, respectively. The empty vectors pEarleygate201-YC and pEarleygate201-YN were used as negative controls. Then, the infiltrated leaves were harvested and then subjected to confocal microscopic analysis at 2 days after agroinfiltration.

**Semi-quantitative RT-PCR to detect the virus induced up-regulation of NbPdc1 and NbAdh1 mRNAs in *N. benthamiana***. To determine the mRNA levels of NbPdc1 and NbAdh1 after tombusvirus infection, *N. benthamiana* leaves were either sap inoculated with TBSV, CIRV or mock inocula or agroinfiltrated with pGD-CNV^20KSTOP^ or pGD-CIRV or pGD-empty. In case of TBSV, both the inoculated and the systemic leaves were harvested at 1.5 days and 4 days after virus infection, respectively. In the case of CIRV and CNV, inoculated leaves were harvested at 2.5 days after virus infection. In case of TBSV p33 and CIRV p36 expression via agroinfiltration, samples were harvested at 3 days post agroinfiltration [7]. NbPdc1 and NbAdh1 mRNA levels were determined by semi-quantitative RT-PCR with primers oligo-d(T) (for RT), #5847 and #5850 (for PCR to detect NbPdc1 mRNA), #7911 and #7966 (for PCR to detect NbAdh1) and #2859 and #2860 (for PCR to detect tubulin mRNA as an internal reference control).

In the case of BaMV, inoculated and systemic leaves were harvested at 3 days and 7 days after virus infection. NbPdc1 and NbAdh1 mRNA were determined by semi-quantitative RT-PCR with primers oligo-d(T) (for RT), primers #5847 and #5850 (for PCR to detect NbPdc1), primers #7911 and #7966 (for PCR to detect NbAdh1), primers #2859 and #2860 (for PCR to detect tubulin mRNA as an internal reference control), and BaMV-CP-F and BaMV-CP-R (for PCR to detect BaMV genomic RNA).

To analyze Pdc1 and Adh1 mRNA levels during TMV infection, *N. benthamiana* leaves were agroinfiltrated with TMV-based vector pJL-36 and plant samples were harvested 2 days after infection from the inoculated leaves and 5 days after infection from the systemic leaves. NbPdc1 and NbAdh1 mRNA levels were measured by semi-quantitative RT-PCR with primers #5847 and #5850 and primers #7911 and #7966, respectively, whereas the tubulin mRNA level was measured as an internal control with #2859 and #2860 oligos.

**Purification of tombusvirus replicase from yeast and in vitro RdRp assay.** To purify TBSV replicase from yeast, plasmids HpGBK-CUP1-Flag-p33, LpGAD-CUP1-Flag-p92 and UpCM189-Tet-DI72 were transformed into BY4741, pdc5∆ and GAL1::PDC1 pdc5∆ yeast strains. The transformed yeasts were pre-grown in SC-ULH^−^ medium supplemented with 2% galactose at 23°C for 16 h. Then, individual yeast cultures were resuspended in SC-ULH^−^ medium containing 50 μM CuSO_4_ and supplemented with 2% galactose or 2% glucose at 23°C for 16 h. TBSV replicase preparations were affinity-purified as described earlier [1,8]. In vitro RdRp activity assay was performed in 20 μl total volume containing 0.5 μg DI-72 region I/III (-)RNA or SL123(+)RNA, 2 μl Flag-affinity purified replicase, 2 μl of 1 M Tris-HCl (pH 8.0), 0.5 μl 1M Tris-HCl (pH 9.0), 0.5 μl 1M MgCl_2_, 0.5 μl of 1 M DTT, 5 μl of 10 mM ATP, CTP, and GTP and 0.1 mM UTP and 0.2 μl of ^32^P-UTP. Reaction mixtures were incubated for 3 h at 25°C, followed by phenol/chloroform extraction and isopropanol/ammonium acetate (10:1) precipitation. ^32^P-UTP-labeled RNA products were analyzed in 5% acrylamide/8M urea gels [9,10].

**In vitro RdRp activation assay.** To obtain the soluble (supernatant) fraction used for the *in vitro* RdRp activation assay, the CFEs prepared from BY4741, pdc5∆ and GAL1::PDC1 pdc5∆ yeast strains were centrifuged at 4°C for 20 min at 42,000 × g. The RdRp activation assay was conducted in 20 μl total volume containing 2 μl soluble fraction of CFEs, 0.5 μg MBP-p92Δ167N, 0.5 µg (+)DI-mini RNA template, 30 mM HEPES-KOH, pH 7.4, 150 mM potassium acetate, 5 mM magnesium acetate, 0.13 M sorbitol, 0.2 μl actinomycin D (5 mg/ml), 2 μl of 150 mM creatine phosphate, 0.2 μl of 10 mg/ml creatine kinase, 0.2 μl of RNAse inhibitor, 0.2 μl of 1 M dithiothreitol (DTT), 2 μl of 10 mM ATP, CTP, and GTP and 0.1 mM UTP and 0.2 μl of ^32^P-UTP. Reaction mixtures were incubated for 3 h at 25°C, followed by phenol/chloroform extraction and isopropanol/ammonium acetate (10:1) precipitation. ^32^P-UTP-labeled RNA products were analyzed in 5% acrylamide/8 M urea gels [9,10].

**Plasmids described in previous studies.** The references for the plasmids listed in S2 Table are described in the following publications: [4,5,8,11-14].

**References:**

1. Panaviene Z, Panavas T, Serva S, Nagy PD (2004) Purification of the cucumber necrosis virus replicase from yeast cells: role of coexpressed viral RNA in stimulation of replicase activity. J Virol 78: 8254-8263.

2. Panavas T, Nagy PD (2003) Yeast as a model host to study replication and recombination of defective interfering RNA of Tomato bushy stunt virus. Virology 314: 315-325.

3. Mendu V, Chiu M, Barajas D, Li Z, Nagy PD (2010) Cpr1 cyclophilin and Ess1 parvulin prolyl isomerases interact with the tombusvirus replication protein and inhibit viral replication in yeast model host. Virology 406: 342-351.

4. Li Z, Barajas D, Panavas T, Herbst DA, Nagy PD (2008) Cdc34p ubiquitin-conjugating enzyme is a component of the tombusvirus replicase complex and ubiquitinates p33 replication protein. J Virol 82: 6911-6926.

5. Li Z, Pogany J, Panavas T, Xu K, Esposito AM, et al. (2009) Translation elongation factor 1A is a component of the tombusvirus replicase complex and affects the stability of the p33 replication co-factor. Virology 385: 245-260.

6. Imamura H, Nhat KP, Togawa H, Saito K, Iino R, et al. (2009) Visualization of ATP levels inside single living cells with fluorescence resonance energy transfer-based genetically encoded indicators. Proc Natl Acad Sci U S A 106: 15651-15656.

7. Jaag HM, Nagy PD (2009) Silencing of Nicotiana benthamiana Xrn4p exoribonuclease promotes tombusvirus RNA accumulation and recombination. Virology 386: 344-352.

8. Chuang C, Prasanth KR, Nagy PD (2017) The Glycolytic Pyruvate Kinase Is Recruited Directly into the Viral Replicase Complex to Generate ATP for RNA Synthesis. Cell Host Microbe 22: 639-652 e637.

9. Pogany J, Stork J, Li Z, Nagy PD (2008) In vitro assembly of the Tomato bushy stunt virus replicase requires the host Heat shock protein 70. Proc Natl Acad Sci U S A 105: 19956-19961.

10. Pogany J, Nagy PD (2008) Authentic replication and recombination of Tomato bushy stunt virus RNA in a cell-free extract from yeast. J Virol 82: 5967-5980.

11. Barajas D, Li Z, Nagy PD (2009) The Nedd4-type Rsp5p ubiquitin ligase inhibits tombusvirus replication by regulating degradation of the p92 replication protein and decreasing the activity of the tombusvirus replicase. J Virol 83: 11751-11764.

12. Xu K, Nagy PD (2016) Enrichment of Phosphatidylethanolamine in Viral Replication Compartments via Co-opting the Endosomal Rab5 Small GTPase by a Positive-Strand RNA Virus. PLoS Biol 14: e2000128.

13. Xu K, Lin JY, Nagy PD (2014) The hop-like stress-induced protein 1 cochaperone is a novel cell-intrinsic restriction factor for mitochondrial tombusvirus replication. J Virol 88: 9361-9378.

14. Lin W, Wang L, Yan W, Chen L, Chen H, et al. (2017) Identification and characterization of Bamboo mosaic virus isolates from a naturally occurring coinfection in Bambusa xiashanensis. Arch Virol 162: 1335-1339.
