# Supplementary material for: Co-opting the fermentation pathway for tombusvirus replication: Compartmentalization of cellular metabolic pathways for rapid ATP generation
Source: PLoS Pathog. 2019 Oct 24;15(10):e1008092. doi: 10.1371/journal.ppat.1008092 (PMC6830812; doi:10.1371/journal.ppat.1008092)
Supplement: S1 Table — (DOCX) [file ppat.1008092.s008.docx]

**S1 Table**

| **Plasmids constructed in this study** | | | | | | |
| --- | --- | --- | --- | --- | --- | --- |
| No. | Plasmid name | insert source | insert RE digestion sites | primers used in PCR for insert  amplification | plasmid source | plasmid RE digestion sites |
| 1 | UpCM189-Tet-HisPdc1 | pYES-NT-Pdc1 | BamHI and PstI | #5621 and #7564 | pCM189-Tet-His p33 | BamHI and PstI |
| 2 | UpCM189-Tet-Pdc1 | pCM189-Tet-His pdc1 | BamHI and PstI | #5621 and #7564 | pCM189 | BamHI and PstI |
| 3 | UpYES-NT-HisPdc1^S455F^ | pYES-NT-Pdc1 | BamHI and XhoI | 5621/7554/7555/7383 | pYES-NT | BamHI and XhoI |
| 4 | UpCM189-Tet-HisPdc1^S455F^ | UpYES-NT-HisPdc1^S455F^ | BamHI and PstI | #5621 and #7564 | pCM189-Tet-His p33 | BamHI and PstI |
| 5 | UpYES-HisAdh1 | yeast genomic DNA | BamHI and XhoI | #7574 and #7575 | pYES-NT | BamHI and XhoI |
| 6 | UpYES-HisAdh2 | yeast genomic DNA | BglII and XhoI | #7576 and #7575 | pYES-NT | BamHI and XhoI |
| 7 | UpYES-HisAdh3 | yeast genomic DNA | BamHI and XhoI | #7577 and #7578 | pYES-NT | BamHI and XhoI |
| 8 | UpYES-HisAdh4 | yeast genomic DNA | BamHI and XhoI | #7579 and #7580 | pYES-NT | BamHI and XhoI |
| 9 | UpYES-HisAdh5 | yeast genomic DNA | BamHI and XhoI | #7581 and #7582 | pYES-NT | BamHI and XhoI |
| 10 | pPR-N-RE-AtPdc1 | pGD-35S-AtPdc1 | BamHI and SalI | #7501 and #7689 | pPRN-RE | BamHI and SalI |
| 11 | pPR-N-RE-Pdc1 | yeast genomic DNA | BamHI and XhoI | #5621 and #6466 | pPRN-RE | BamHI and SalI |
| 12 | pPR-N-RE-AtAdh1 | A. thaliana cDNA | BamHI and XhoI | #7907 and #7908 | pPRN-RE | BamHI and SalI |
| 13 | pPR-N-RE-Adh1 | pYES-NT-ADH1 | BamHI and XhoI | #7574 and #7575 | pPRN-RE | BamHI and SalI |
| 14 | pPR-N-RE-Adh2 | pYES-NT-ADH2 | BglII and XhoI | #7576 and #7575 | pPRN-RE | BamHI and SalI |
| 15 | pPR-N-RE-Adh3 | pYES-NT-ADH3 | BamHI and XhoI | #7577 and #7578 | pPRN-RE | BamHI and SalI |
| 16 | pPR-N-RE-Adh4 | pYES-NT-ADH4 | BamHI and XhoI | #7579 and #7580 | pPRN-RE | BamHI and SalI |
| 17 | pPR-N-RE-Adh5 | pYES-NT-ADH5 | BamHI and XhoI | #7581 and #7582 | pPRN-RE | BamHI and SalI |
| 18 | UpYES-HisPdc1 | yeast genomic DNA | BamHI and XhoI | #5621 and #7383 | pYES-NT | BamHI and XhoI |
| 19 | UpYES-HisAtPdc1 | pGD-35S-AtPdc1 | BamHI and SalI | #7501 and #7504 | pYES-NT | BamHI and XhoI |
| 20 | UpYES-HisAtAdh1 | A. thaliana cDNA | BamHI and XhoI | #7907 and #7908 | pYES-NT | BamHI and XhoI |
| 21 | pGD-AtPdc1 | A. thaliana cDNA | BamHI and SalI | #7501 and #7502 | pGD-35S | BamHI and XhoI |
| 22 | pGD-HA-AtPdc1 | A. thaliana cDNA | BamHI and SalI | #7573 and #7502 | pGD-35S | BamHI and XhoI |
| 23 | pGD-GFP-HA | HpESC-GFP-TBSVp33/Gal-DI72 | BamHI and XhoI | #6511 and #3712 | pGD-35S | BamHI and XhoI |
| 24 | pGD-T33-Flag | HpESC-GFP-TBSVp33/Gal-DI72 | BglII and XhoI | #4000 and #7834 | pGD-35S | BamHI and XhoI |
| 25 | pGEX-His-RE-AtPdc1 | pGD-35S-AtPdc1 | BamHI and SalI | #7501 and #7502 | pGEX-His--RE | BamHI and XhoI |
| 26 | pGEX-His-RE-AtAdh1 | A. thaliana cDNA | BamHI and XhoI | #7907 and #7908 | pGEX-His--RE | BamHI and XhoI |
| 27 | pGEX-His-RE-Adh1 | pYES-NT-ADH1 | BamHI and XhoI | #7574 and #7575 | pGEX-His--RE | BamHI and XhoI |
| 28 | pMALc-2X-AtPdc1 | pGD-35S-AtPdc1 | BamHI and SalI | #7501 and #7503 | pMALC-2X | BamHI and XhoI |
| 29 | pGD-RFP-AtPdc1 | A. thaliana cDNA | BamHI and SalI | #7501 and #7502 | pGD-N-RFP | BamHI and XhoI |
| 30 | pGD-BFP-AtAdh1 | A. thaliana cDNA | BamHI and XhoI | #7907 and #7908 | pGD-N-BFP | BamHI and SalI |
| 31 | pGD-nYFP-AtPdc1 | A. thaliana cDNA | BamHI and SalI | #7501 and #7502 | pGD-nYFP-MBP | BamHI and SalI |
| 32 | pGD-nYFP-AtAdh1 | A. thaliana cDNA | BamHI and XhoI | #7907 and #7908 | pGD-nYFP-MBP | BamHI and SalI |
| 33 | pGD-GFP-AtPdc1 | pGD-AtPdc1-FLAG | BamHI and SalI | #7501 and #7884 | pGD-N-GFP | BamHI and SalI |
| 34 | pGD-GFP-AtAdh1 | pYES-NT-AtADH1 | BamHI and XhoI | #7907 and #7908 | pGD-N-GFP | BamHI and SalI |
| 35 | TRV2-NbPdc1 | N. benthamiana cDNA | BamHI and XhoI | #5847 and #5848 | TRV2-empty | BamHI and XhoI |
| 36 | TRV2-NbAdh1 | N. benthamiana cDNA | BamHI and XhoI | #7911 and #7912 | TRV2-empty | BamHI and XhoI |
| 37 | pEarleygate201-YC-BaMV-ORF1-capping | pCAMBaMV-S | Gateway BP/LR | 221-BaMV-cap-F/R | pEarleygate201-YC | Gateway BP/LR |
| 38 | pEarleygate201-YC-BaMV-ORF1-Helicase | pCAMBaMV-S | Gateway BP/LR | 221-BaMV-heli-F/R | pEarleygate201-YC | Gateway BP/LR |
| 39 | pEarleygate201-YC-BaMV-ORF1-RdRp | pCAMBaMV-S | Gateway BP/LR | 221-BaMV-rdrp-F/R | pEarleygate201-YC | Gateway BP/LR |
| 40 | pEarleygate201-YC-BaMV-TGBp1 | pCAMBaMV-S | Gateway BP/LR | 221-BaMV-TGBp1-F/R | pEarleygate201-YC | Gateway BP/LR |
| 41 | pEarleygate201-YC-BaMV-TGBp2 | pCAMBaMV-S | Gateway BP/LR | 221-BaMV-TGBp2-F/R | pEarleygate201-YC | Gateway BP/LR |
| 42 | pEarleygate201-YC-BaMV-TGBp3 | pCAMBaMV-S | Gateway BP/LR | 221-BaMV-TGBp3-F/R | pEarleygate201-YC | Gateway BP/LR |
| 43 | pEarleygate201-YC-BaMV-CP | pCAMBaMV-S | Gateway BP/LR | 221-BaMV-CP-F/R | pEarleygate201-YC | Gateway BP/LR |
| 44 | pEarleygate201-YN-AtPdc1 | A. thaliana cDNA | Gateway BP/LR | 221-AtPdc1-F/R | pEarleygate201-YN | Gateway BP/LR |
| 45 | pEarleygate201-YN-AtAdh1 | A. thaliana cDNA | Gateway BP/LR | 221-AtAdh1-F/R | pEarleygate201-YN | Gateway BP/LR |
