# Supplementary material for: Co-opting the fermentation pathway for tombusvirus replication: Compartmentalization of cellular metabolic pathways for rapid ATP generation
Source: PLoS Pathog. 2019 Oct 24;15(10):e1008092. doi: 10.1371/journal.ppat.1008092 (PMC6830812; doi:10.1371/journal.ppat.1008092)
Supplement: S2 Table — (DOCX) [file ppat.1008092.s009.docx]

**S2 Table**

| Plasmids described in previous studies | | |
| --- | --- | --- |
| No. | Plasmid name | Source (see references in S1 Text) |
| 1 | HpGBK-CUP1-Hisp33/ADH-DI72 | Dr.Chingkai Chuang(University of Kentucky) |
| 2 | HpGBK-Gal-HisT33/Gal-DI72 | Dr. Kai Xu(University of Kentucky) |
| 3 | LpGAD-Gal-HisT92 | Dr. Kai Xu(University of Kentucky) |
| 4 | HpGBK-CUP1-Hisp33/Gal-DI72 | [11] Barajas et al., 2009 |
| 5 | LpGAD-CUP1-Hisp92 | [11] Barajas et al., 2009 |
| 6 | HpESC-CUP1-Hisp36/Gal-DI72 | Dr. J. Pogany(University of Kentucky) |
| 7 | LpESC-CUP1-Hisp95 | Dr. J. Pogany(University of Kentucky) |
| 8 | pESC-His/Cup/FHV/RNA1/Frameshift/TRSVRRZ | Dr. J. Pogany(University of Kentucky) |
| 9 | pGAD-Leu/Cup/FHV/Protein-A/C-term/HA/FLAG | Dr. J. Pogany(University of Kentucky) |
| 10 | pGAD-BT2-N-His33 | [4] Li et al., 2008 |
| 11 | pPR-N-RE-ssa1 | [5] Li et al., 2009 |
| 12 | HpGBK-CUP1-Flagp33/Gal-DI72 | [11] Barajas et al., 2009 |
| 13 | HpGBK-CUP1-Flagp33 | Miss Molho(Univrsity of Kentucky) |
| 14 | LpGAD-CUP1-Flag92 | [11] Barajas et al., 2009 |
| 15 | HpGBK-CUP1-Flagp36/Gal-DI72 | Dr. J. Pogany (U. Kentucky) |
| 16 | LpGAD-CUP1-Flag95 | Dr. J. Pogany (U. Kentucky) |
| 17 | UpCM189-TET-DI72 | Miss Molho(Univrsity of Kentucky) |
| 18 | pGD-35S-p19 | [12] Xu and Nagy, 2016 |
| 19 | pMALc-2X-T33C | [12] Xu and Nagy, 2016 |
| 20 | pMALc-2X-C36C | [12] Xu and Nagy, 2016 |
| 21 | pGD-35S-RFP-T33 | [12] Xu and Nagy, 2016 |
| 22 | pGD-35S-RFP-C36 | [12] Xu and Nagy, 2016 |
| 23 | pGD-35S-GFP-SKL | [12] Xu and Nagy, 2016 |
| 24 | pGD-35S-GFP-AtTim21 | [12] Xu and Nagy, 2016 |
| 25 | pGD-35S-T33-cYFP | [12] Xu and Nagy, 2016 |
| 26 | pGD-35S-C36-cYFP | [12] Xu and Nagy, 2016 |
| 27 | pGD-35S-C-cYFP | [12] Xu and Nagy, 2016 |
| 28 | pGD-35S-nYFP-MBP | [12] Xu and Nagy, 2016 |
| 29 | pGD-35S-RFP-SKL | [12] Xu and Nagy, 2016 |
| 30 | pGD-35S-RFP-AtTim21 | [12] Xu and Nagy, 2016 |
| 31 | pGD-p33 | Dr.Barajas(University of Kentucky) |
| 32 | pGD-p92 | Dr.Barajas(University of Kentucky) |
| 33 | pGD-p36 | Dr.Barajas(University of Kentucky) |
| 34 | pGD-p95 | Dr.Barajas(University of Kentucky) |
| 35 | pGD-DI72 | Dr.Barajas(University of Kentucky) |
| 36 | TRV-cGFP | [13] Xu et al., 2014 |
| 37 | pGD-MS2CP-RFP | Mr.Cheng-Yu Wu(U. Kentucky) |
| 38 | pGD-(+)DI72-MS2hp | Mr.Cheng-Yu Wu(U. Kentucky) |
| 39 | pGD-(-)DI72-MS2hp | Mr.Cheng-Yu Wu(U. Kentucky) |
| 40 | LpGAD-ADH-ATeam^YEMK^-p92 | [8] Chuang et al., 2017 |
| 41 | LpGAD-ADH-ATeam^RK^-p92 | [8] Chuang et al., 2017 |
| 42 | pGD-p33-ATeam^YEMK^ | [8] Chuang et al., 2017 |
| 43 | pGD-p36-ATeam^YEMK^ | [8] Chuang et al., 2017 |
| 44 | pMALc-2X-Pdc1 | Dr.Chingkai Chuang(University of Kentucky) |
| 45 | pCAMBaMV-S | [14] Lin et al., 2017 |
