# Supplementary material for: Co-opting the fermentation pathway for tombusvirus replication: Compartmentalization of cellular metabolic pathways for rapid ATP generation
Source: PLoS Pathog. 2019 Oct 24;15(10):e1008092. doi: 10.1371/journal.ppat.1008092 (PMC6830812; doi:10.1371/journal.ppat.1008092)
Supplement: S3 Table — (DOCX) [file ppat.1008092.s010.docx]

**S3 Table**

| **Primers used in this study** | | |
| --- | --- | --- |
| NO. | Name | Sequence(5' to 3') |
| 1 | 2859/tubulin/Nb1283/T7/R | TAATACGACTCACTATAGGAACCAAATCATTCATGTTGCTCTC |
| 2 | 2860/tubulin/Nb1080/F | TAGTGTATGTGATATCCCACCAA |
| 3 | 3712/HA/rsGFP/XhoI/R | CGGCCTCGAGTTACGCATAGTCAGGAACATCGTATGGGTAGAGTCCGGACTTGTATAGTT |
| 4 | 4000/TBSV33/Bgl/F | CCAGAGATCTATGGAGACCATCAAGAGAATG |
| 5 | 5621/ScPDC1/BamHI/F | CGCCGGATCCATGTCTGAAATTACTTTGGGTAAATA |
| 6 | 5847/NbPDC1-S-BamHI-F1 | CGGGATCCGGCGTCACCGACGTTTTCG |
| 7 | 5848/NbPDC1-S-XhoI-R1 | CCGCTCGAGCCAATAGTATGATGAAGGATTCTG |
| 8 | 5850/NbPDC1-S-XhoI-R2 | CCGCTCGAGGGCATCACTGCAACAGCATATC |
| 9 | 6466/PDC1/ctHis/XhoI/R | CGCGCTCGAGTTAATGGTGATGGTGATGATGTTGCTTAGCGTTGGTAGC |
| 10 | 6511/rsGFP/ATG/BamHI/F | CGGGATCCATGGGTAAAGGAGAAGAACTTTTCACTGG |
| 11 | 7383/ScPDC1/Xho1/TAG/Xba1/R | CTAGTCTAGACTACTCGAGTTGCTTAGCGTTGGTAGCAGC |
| 12 | 7475/ScPDC2-S1/F | AACACCAAGCAGTAAAGAGACAGCTTTATTATAACCAGCATGCGTACGCTGCAGGTCGAC |
| 13 | 7476/ScPDC2-S4/R | CCGCCATTAGACAAATATTATATCTTTGCTGAATGGAAAGCATCGATGAATTCTCTGTCG |
| 14 | 7501/AtPDC1/BamHI/F | ACGCGGATCCATGGACACCAAAATCGGATCGATC |
| 15 | 7502/AtPDC1/SalI/R | ACGCGTCGACCTACTGAGGATTGGGAGGACGG |
| 16 | 7504/Pdc5-K/O mutant/S1/F | TCAAAGAGA ACAACACAAT ACAATAACAA GAAGAACAAAATGCGTACGCTGCAGGTCGAC |
| 17 | 7505/Pdc5-K/O mutant/S2/R | TACACAAACGTTGAATCATGAGTTTTATGTTAATTAGCTTAATCGATGAATTCGAGCTCG |
| 18 | 7554/ScPDC1/S455F/F | GCAATTGACTGTTCAAGAAATCTTCACCATGATCAGATGG |
| 19 | 7555/ScPDC1/S455F/R | CCATCTGATCATGGTGAAGATTTCTTGAACAGTCAATTGC |
| 20 | 7562/Pdc1-GAL mutant-long/S1/F | CTCAATTATTATTTTCTACTCATAACCTCACGCAAAATAACACAGTCAAATCAATCAAAATGCGTACGCTGCAGGTCGAC |
| 21 | 7563/Pdc1-GAL mutant-long/S4/R | GGTGTTAACGTTGACTTGCTTTAATCTTTCGAACAAATATTTACCCAAAGTAATTTCAGACATCGATGAATTCTCTGTCG |
| 22 | 7564/ScPdc1/stop/Pst1/R | CGAACTGCAGTTATTGCTTAGCGTTGGTAGCAGC |
| 23 | 7573/AtPDC1/BamHI/HA/F | CGCGGATCCATGTACCCATACGATGTTCCAGATTACGCTGACACCAAAATCGGATCGATC |
| 24 | 7574/ScADH1/BamHI/F | ACGCGGATCCATGTCTATCCCAGAAACTCAAAAAGG |
| 25 | 7575/ScADH1/XhoI/R | ACCGCTCGAGTTATTTAGAAGTGTCAACAACGTATCTACC |
| 26 | 7576/ScADH2/BglII/F | AGGAAGATCTATGTCTATTCCAGAAACTCAAAAAGC |
| 27 | 7577/ScADH3/BamHI/F | ACGCGGATCCATGTTGAGAACGTCAACATTGTTCAC |
| 28 | 578/ScADH3/XhoI/R | ACCGCTCGAGTTATTTACTAGTATCGACGACGTATCTACCC |
| 29 | 7579/ScADH4/BamHI/F | ACGCGGATCCATGTCTTCCGTTACTGGGTTTTAC |
| 30 | 7580/ScADH4/XhoI/R | ACCGCTCGAGTTAATATTCATAGGCTTTCTTGATAATGG |
| 31 | 7581/ScADH5/BamHI/F | ACGCGGATCCATGCCTTCGCAAGTCATTCCTG |
| 32 | 7582/ScADH5/XhoI/R | ACCGCTCGAGTCATTTAGAAGTCTCAACAACATATCTACC |
| 33 | 7689/AtPDC1/HA/SalI/R | ACGCGTCGACCTAAGCGTAATCTGGAACATCGTATGGGTACTGAGGATTGGGAGGACGG |
| 34 | 7834/TBSVp33/Flag/XhoI/R | ACCGCTCGAGCTACTTGTCATCGTCGTCCTTGTAGTCTTTGACACCCAGGGACTCCT |
| 35 | 7907/AtADH1/BamHI/F | ACGCGGATCCATGTCTACCACCGGACAGATTATTC |
| 36 | 7908/AtADH1/stop/XhoI/R | ACCGCTCGAGTCAAGCACCCATGGTGATGATGC |
| 37 | 7909/NbADH1/VIGS-S1/BamHI/F | ACGCGGATCCCGTTGCAAAGCTGCGATTGC |
| 38 | 7911/NbADH1/VIGS-S2/BamHI/F | ACGCGGATCCCCTACCAAAGGTTCTACTGTAGCTA |
| 39 | NbADH1/VIGS-S2/XhoI/R | ACCGCTCGAGCCCAACCATCATGAACACATTC |
| 40 | 7966/NbADH1-mRNA/R | TCAATGTCCCATGGTGATCATG |
| 41 | 221-BaMV-cap-F | GGGGACAAGTTTGTACAAAAAAGCAGGCTTCATGGCACTCGTTTCTAAAGTCTTTG |
| 42 | 221-BaMV-cap-R | GGGGACCACTTTGTACAAGAAAGCTGGGTCTTCGGTAATTGCTGCGTCTGTG |
| 43 | 221-BaMV-heli-F | GGGGACAAGTTTGTACAAAAAAGCAGGCTTCATGAGCAAAGAGCGGAAGTGCC |
| 44 | 221-BaMV-heli-R | GGGGACCACTTTGTACAAGAAAGCTGGGTCGTGTTCCTTTGTAAGGTTGAGGATG |
| 45 | 221-BaMV-rdrp-F | GGGGACAAGTTTGTACAAAAAAGCAGGCTTCATGGTCACCCACATTGCCGTGGA |
| 46 | 221-BaMV-rdrp-R | GGGGACCACTTTGTACAAGAAAGCTGGGTCACTAGAGAATAAACCTTCTTCGCCA |
| 47 | 221-BaMV-TGBp1-F | GGGGACAAGTTTGTACAAAAAAGCAGGCTTCATGGATAACCGGATAACTGACCTAC |
| 48 | 221-BaMV-TGBp1-R | GGGGACCACTTTGTACAAGAAAGCTGGGTCGGTGGTCTGGCCAGATGAAGAG |
| 49 | 221-BaMV-TGBp2-F | GGGGACAAGTTTGTACAAAAAAGCAGGCTTCATGGACCAGCCTCTTCATCTGG |
| 50 | 221-BaMV-TGBp2-R | GGGGACCACTTTGTACAAGAAAGCTGGGTCGCATGGTGGGTGATTCCGGT |
| 51 | 221-BaMV-TGBp3-F | GGGGACAAGTTTGTACAAAAAAGCAGGCTTCATGCTAAACACTGACACACTATGC |
| 52 | 221-BaMV-TGBp3-R | GGGGACCACTTTGTACAAGAAAGCTGGGTCGCTGGAGGTGGTGTGGTAGCAG |
| 53 | 221-BaMV-CP-F | GGGGACAAGTTTGTACAAAAAAGCAGGCTTCATGTCTGGAGCTGGAACGGGAAC |
| 54 | 221-BaMV-CP-R | GGGGACCACTTTGTACAAGAAAGCTGGGTCGTCTGATGTTGGTTCGGGAAG |
| 55 | 221-AtPdc1-F | GGGGACAAGTTTGTACAAAAAAGCAGGCTTCATGGACACCAAAATCGGATCGATC |
| 56 | 221-AtPdc1-R | GGGGACCACTTTGTACAAGAAAGCTGGGTCCTGAGGATTGGGAGGACGG |
| 57 | 221-AtAdh1-F | GGGGACAAGTTTGTACAAAAAAGCAGGCTTCATGTCTACCACCGGACAGATTATTC |
| 58 | 221-AtAdh1-R | GGGGACCACTTTGTACAAGAAAGCTGGGTCAGCACCCATGGTGATGATGC |
| 59 | qPCR-EF1a-1-F | TGGTGTCCTCAAGCCTGGTATG |
| 60 | qPCR-EF1a-1-R | TGAGATCCTTAACCGCAACATTC |
| 61 | qPCR-BaMV-F | CAGCATCACCGACCCATC |
| 62 | qPCR-BaMV-R | GCGTACCGTTCTAGCGTGTT |
| 63 | BaMV-CP-F | ATGTCTGGAGCTGGAACGGGAA |
| 64 | BaMV-CP-R | GTCTGATGTTGGTTCGGGAAG |
